# Exploring mitophagy levels in *Drosophila* Malpighian tubules unveils the pivotal role of mitophagy in kidney function and diabetic kidney disease

**DOI:** 10.1038/s12276-025-01558-2

**Published:** 2025-10-23

**Authors:** Kang-Min Lee, Jihun Kim, Hye Lim Jung, Young Yeon Kim, Jihoon Lee, Yeon-Ju Lee, Eunhee Yoo, Hyi-Seung Lee, Jeanho Yun

**Affiliations:** 1 Department of Biochemistry, College of Medicine, Dong-A University, Busan, Republic of Korea; 2 Department of Translational Biomedical Sciences, Graduate School of Dong-A University, Busan, Republic of Korea; 3 Peripheral Neuropathy Research Center, Dong-A University, Busan, Republic of Korea; 4 Korea Institute of Ocean Science and Technology, Busan, Republic of Korea; 5 Altmedical Co. Ltd, Seoul, Republic of Korea

**Keywords:** Mitophagy, End-stage renal disease

## Abstract

Mitophagy has been implicated in kidney function and related diseases. However, a direct analysis of mitophagy in kidney models, including disease models, remains notably lacking. Here we analyzed mitophagy levels in *Drosophila* Malpighian tubules, a functional analog of the human kidney, using a transgenic model of the engineered mitophagy reporter mt-Keima. We found that mitophagy is highly active in the major cell types of the Malpighian tubules, including renal stem cells, principal cells and stellate cells. Notably, the suppression of mitophagy by genetic downregulation of mitophagy-related genes, such as ATG5 and ULK1, led to a significant decrease in the secretion function of the Malpighian tubules, suggesting that mitophagy is essential for their proper function. Interestingly, a continuous high-sugar diet, which is used as a model for diabetic kidney disease, caused a reduction in mitophagy levels in principal cells before the development of mitochondrial dysfunction and defective secretion. Importantly, stimulation of mitophagy with the recently developed mitophagy inducer PDE701 rescued both mitochondrial dysfunction and defective phenotypes in a diabetic kidney disease model. Our results highlight the pivotal role of mitophagy in kidney function and suggest that modulating mitophagy could be a potential strategy for treating kidney diseases.

## Mitophagy vital for kidney function and disease management

Mitophagy is a process that removes damaged mitochondria to keep cells healthy. This study looks at how mitophagy works in the kidneys using fruit flies, which have similar kidney-like structures called Malpighian tubules. Here researchers used a special protein called mt-Keima to measure mitophagy in these tubules. They found that mitophagy is crucial for the function of the tubules. When mitophagy was reduced, the tubules did not work well, especially under conditions mimicking diabetic kidney disease (DKD). The study used a high-sugar diet to create a DKD model in flies, which led to decreased mitophagy and kidney dysfunction. However, a new compound called PDE701 increased mitophagy and improved kidney function in these flies. The findings suggest that boosting mitophagy could help treat kidney diseases such as DKD.

This summary was initially drafted using artificial intelligence, then revised and fact-checked by the author.

## Introduction

Mitophagy, a selective removal mechanism for damaged or unnecessary mitochondria via the core autophagic machinery, has emerged as a pivotal process in maintaining cellular homeostasis under normal physiological conditions and stress conditions^1,2^. Extensive research conducted over the past few decades has established the essential role of mitophagy in regulating various cellular processes, including mitochondrial quality control, differentiation and development, cell reprogramming, cell death and the immune response. Furthermore, mitophagy dysfunction has been strongly associated with various human diseases, such as neurodegenerative diseases, cardiovascular diseases, metabolic diseases and kidney diseases^2–6^. For a better understanding of the role of mitophagy in tissue function and human diseases, direct analyses of mitophagy activity in in vivo tissue in normal and disease contexts are essential. Recent developments in mitophagy assay methods, including the mitochondria-targeted Keima (mt-Keima) assay, have enhanced our ability to investigate mitophagy in vivo, but investigations of the precise role of mitophagy in individual tissues and organs are still limited^5,7^.

The kidney is an essential organ for eliminating waste products and regulating internal fluid balance. Mitochondria play crucial roles in kidney function, providing the energy required for essential processes such as waste removal, electrolyte balance and blood pressure regulation^8,9^. As the kidney is a high-energy-demand organ, kidney cells rely heavily on efficient mitochondrial function to maintain cellular health and overall renal performance^9,10^. Mapping evidence has demonstrated that mitochondrial dysfunction is an important contributor to various kidney diseases, including chronic kidney disease (CKD), acute kidney injury and diabetic kidney disease (DKD, also known as diabetic nephropathy)^11–14^. Mitophagy plays an essential role in mitochondrial homeostasis, reducing oxidative stress and inflammation by removing dysfunctional mitochondria, thereby protecting kidney cells and supporting energy metabolism^15–17^. There is increasing evidence that mitophagy plays a critical role in kidney homeostasis and kidney disease, including DKD^18–20^. However, direct measurements of mitophagy activity in kidney tissue and investigations of the role of mitophagy in kidney disease are lacking.

Using a transgenic model of the mt-Keima protein, which is a pH-dependent fluorescent reporter, we previously studied mitophagy in mice and *Drosophila* in vivo^21,22^. In this study, we explored the role of mitophagy in Malpighian tubules, a *Drosophila* functional analog of the mammalian kidney^23–25^. Analysis of mitophagy levels in Malpighian tubules revealed that mitophagy activity was important for maintaining the mitochondrial and secretory functions of Malpighian tubules. Importantly, mitophagy activity was decreased in a DKD model and the stimulation of mitophagy with a recently developed mitophagy inducer ameliorated functional and structural defects in mitochondrial function and DKD symptoms.

## Methods

### Drosophila strains

Fly lines were raised at 25 °C and 50% humidity in a 12 h:12 h light:dark cycle with standard fly media. For experiments, naive male flies were collected at eclosion and aged in groups of 10−15. Flies were transferred to fresh food every 2−3 days. Flies carrying *tubP-Gal80*^*ts*^ were raised at 18 °C until eclosion, and adult flies were aged at 30 °C.

To generate the *UAS-dmt-Keima* line, Keima cDNA from pLESIP-Keima was subcloned into a pACU2 vector. Subsequently, the *Drosophila* COX VIII sequence amplified by genomic PCR using a pair of primers (forward primer 5′-ATGTTCCAAAACAGCGCTG-3′, reverse primer 5′-GGTGGAGATGCGCTGGGTC-3′) and subcloned in front of Keima coding sequence (CDS). The pACU2−dmt−Keima construct was verified for correct sequence and orientation via DNA sequencing. After confirming correct subcloning, the construct DNA was microinjected into a specific site on the second chromosome (VIE-72A) or third chromosome (VIE-49B) using phiC3^26^ via the microinjection services provided by the Korea *Drosophila* Resource Center (KDRC).

The *UAS-mt-Keima*^22^, *UAS-mito-roGFP2-Orp1*^27^ and *UAS-ATG5 RNAi*^28^ lines were obtained from previously reported studies. The UAS-ATG5 RNAi line expresses a double-stranded RNA targeting the autophagy-related gene *ATG5*, which is essential for autophagosome formation. Knockdown was induced in specific tissues via the Gal4-UAS system, leading to RNA interference-mediated suppression of *ATG5* mRNA. *UAS-Parkin RNAi*^29^ was kindly provided by Dr. Jongkyeong Chung of Seoul National University. *w*^*1118*^ (VDRC 60000) and *UAS-AMPKα RNAi* (VDRC 106200) were obtained from the Vienna *Drosophila* Resource Center. *da-Gal4* (BDSC 55851), *c42-Gal4* (BDSC 30835), *tsh-Gal4* (BDSC 3040), *tubP-Gal80*^*ts*^ (BDSC 7019), *UAS-ULK1 RNAi* (BDSC 44034), *UAS-mitoQC* (BDSC 91640), *UAS-mitoHAGFP* (BDSC 8442), *UAS-SOD1* (BDSC 24750) and *UAS-Tor RNAi* (BDSC 34639) were obtained from the Bloomington *Drosophila* Stock Center. *esg-Gal4* (DGRG 112304) was obtained from the Kyoto Stock Center. *UAS-AMPKα-WT* (KDRC 10097) was obtained from KDRC. *UAS-ATG7 RNAi* (5489R-2) was obtained from the National Institute of Genetics.

Detailed descriptions of the imaging-based assays, mitochondrial and physiological analyses, gene expression quantification and *Drosophila* genotypes (Supplementary Table 1) are available in the Supplementary Information.

## Results

### Analysis of mitophagy levels revealed highly active mitophagy in Malpighian tubules

**Fig. 1:**
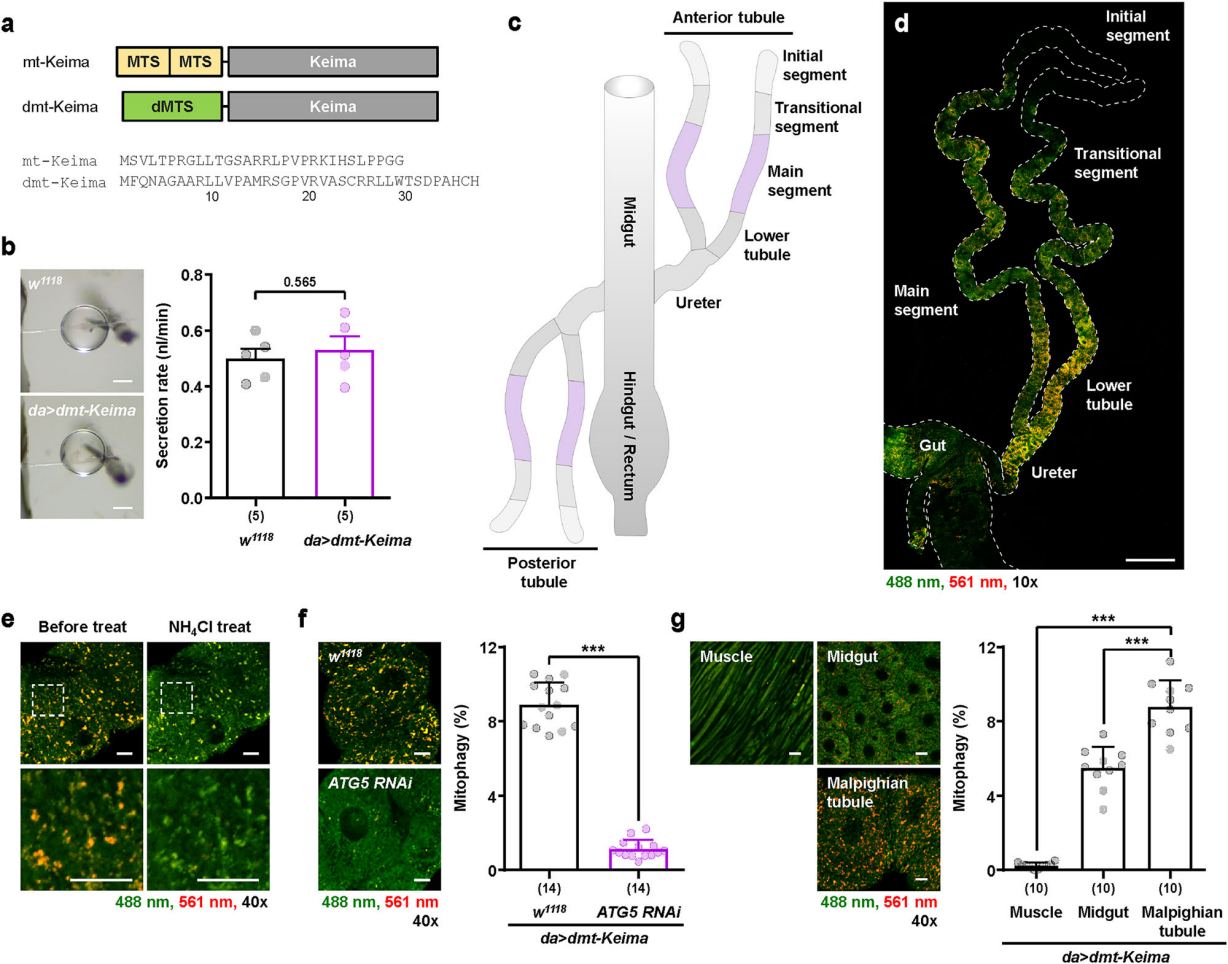
Assessment of mitophagy in dmt-Keima *Drosophila.* **a** Transgenic constructs (top) and amino acid sequences (bottom) for mt-Keima or dmt-Keima. The mitochondrial-targeting sequence (MTS), derived from *Drosophila* Cox8, was used for dmt-Keima. **b** Representative images of fluid droplets from the Malpighian tubules of wild-type (*w*^*1118*^) and dmt-Keima (*da>dmt-Keima*) fly males (left). The fluid secretion rates for the Malpighian tubules from each fly strain (right). The data are shown as mean ± s.e.m., and the secretion rates from 5 biological replicates with 5–8 flies per group were measured. Scale bars, 200 μm. **c**,**d** A structural diagram (**c**) and confocal image (**d**) of Malpighian tubules and guts of male flies carrying *da>dmt-Keima*. The Malpighian tubules of *Drosophila* branch out between the midgut and hindgut and extend in both the anterior and posterior directions. Each branch is composed of the ureter, lower tubule, main segment, transitional segment and initial segment. The dashed white line outlines the anterior Malpighian tubules and gut. Scale bar, 200 μm. **e** The effects of NH_4_Cl treatment on dmt-Keima fluorescence. The main segment of the Malpighian tubules from a male fly carrying *da>dmt-Keima* was treated with NH_4_Cl (50 mM) (top). The boxed regions are presented in an enlarged format for a more detailed view (bottom). Scale bars, 10 μm. **f** Representative dmt-Keima fluorescence images (left) and quantitative analysis of mitophagy (right) in the main segment of Malpighian tubules from control flies (*da>dmt-Keima, w*^*1118*^) or ATG5 RNAi (*da>dmt-Keima, ATG5 RNAi*). The data are shown as mean ± s.d., and the mitophagy levels of *n* = 14 individual flies were quantified. Scale bars, 10 μm. **g** Assessment of mitophagy levels in various fly tissues from adult male flies harboring *da>dmt-Keima*. Representative dmt-Keima fluorescence images (left) and quantitative analysis of mitophagy (right). The data are shown as mean ± s.d. (*n* = 10). Scale bars, 10 μm. dmt-Keima fluorescence was imaged at 488 nm (green) and 561 nm (red); mitophagy levels were quantified based on the red/green fluorescence ratio, which reflects mitochondrial acidification. The 10× and 40× indicate the magnifications of the objective lenses used for confocal imaging. The numbers in parentheses indicate *n*. Significance was determined by Student’s *t-*tests (**b** and **f**) or one-way analysis of variance (ANOVA) with Šidák correction (**g**). The number above the bars is the *P* value. ***P < 0.001.

To investigate mitophagy levels in *Drosophila* Malpighian tubules, we employed a previously established pH-dependent reporter mt-Keima-based mitophagy analysis system^21,22^. We previously developed a method that can quantitatively measure mitophagy levels in tissues through the analysis of mt-Keima fluorescence images and this method was used to analyze the mitophagy activity of various tissues of mice and *Drosophila*^21,22^. To increase the fluorescence signal of mt-Keima in *Drosophila* tissue, we generated an engineered mt-Keima by replacing the human COX XIII mitochondrial-targeting sequence of mt-Keima with the *Drosophila* COX XIII mitochondrial-targeting sequence (hereafter referred to as dmt-Keima, a version of mt-Keima containing a *Drosophila*-specific mitochondrial-targeting sequence) (Fig. 1a). Compared with that of mt-Keima (*UAS-mt-Keima; da-Gal4*), the fluorescence intensity of *Drosophila* tissues expressing dmt-Keima (*UAS-dmt-Keima; da-Gal4*) was significantly greater, whereas the level of mitophagy remained unchanged (Supplementary Fig. 1a–c). Thus, we employed dmt-Keima to investigate the mitophagy level in Malpighian tubules. We have previously shown that the expression of mt-Keima does not directly affect mitochondrial or tissue function in mice or *Drosophila*^22^. Consistently, the fluid secretion rate of Malpighian tubules isolated from *Drosophila* expressing dmt-Keima, as measured via the Ramsay assay^30^, was comparable to that of the control flies (Fig. 1b), indicating that dmt-Keima expression also does not interfere with Malpighian tubule function. When Malpighian tubules expressing dmt-Keima via the *da-Gal4* driver were imaged via 488 nm and 561 nm excitation lasers, multiple red round punctate structures were observable in most regions except for the initial segment at the end of the Malpighian tubules (Fig. 1c, d). We previously demonstrated that red punctate structures represent mitochondria in acidic lysosomal compartments with high 561/488 nm ratios in various *Drosophila* tissues^22^. Consistently, red puncta rapidly changed to a green signal upon treatment with ammonium chloride (NH_4_Cl), a compound that can alkalinize intracellular compartments (Fig. 1e)^31^. Moreover, the knockdown of the essential autophagy gene *ATG5* via the expression of *ATG5* RNAi resulted in a significant reduction in mitophagy in Malpighian tubules (Fig. 1f). These results confirm that the dmt-Keima-based mitophagy assay yields reliable measurements of mitophagy levels in Malpighian tubules, similar to other tissues in *Drosophila* and mice^21,22^. The results of the quantitative analysis revealed that the mitophagy levels in the flight muscle and midgut were consistent with those reported in previous studies^22,32^ (Fig. 1g). Interestingly, the mitophagy level in the Malpighian tubules was significantly higher than that in the flight muscle and was 1.6 times higher than that in the midgut (Fig. 1g). Analyses of mitophagy levels via another mitophagy reporter, mitoQC^33^, also revealed similar results (Supplementary Fig. 1d), further confirming highly active mitophagy in Malpighian tubules.

### Mitophagy levels in major cell types vary depending on their location in Malpighian tubules

**Fig. 2:**
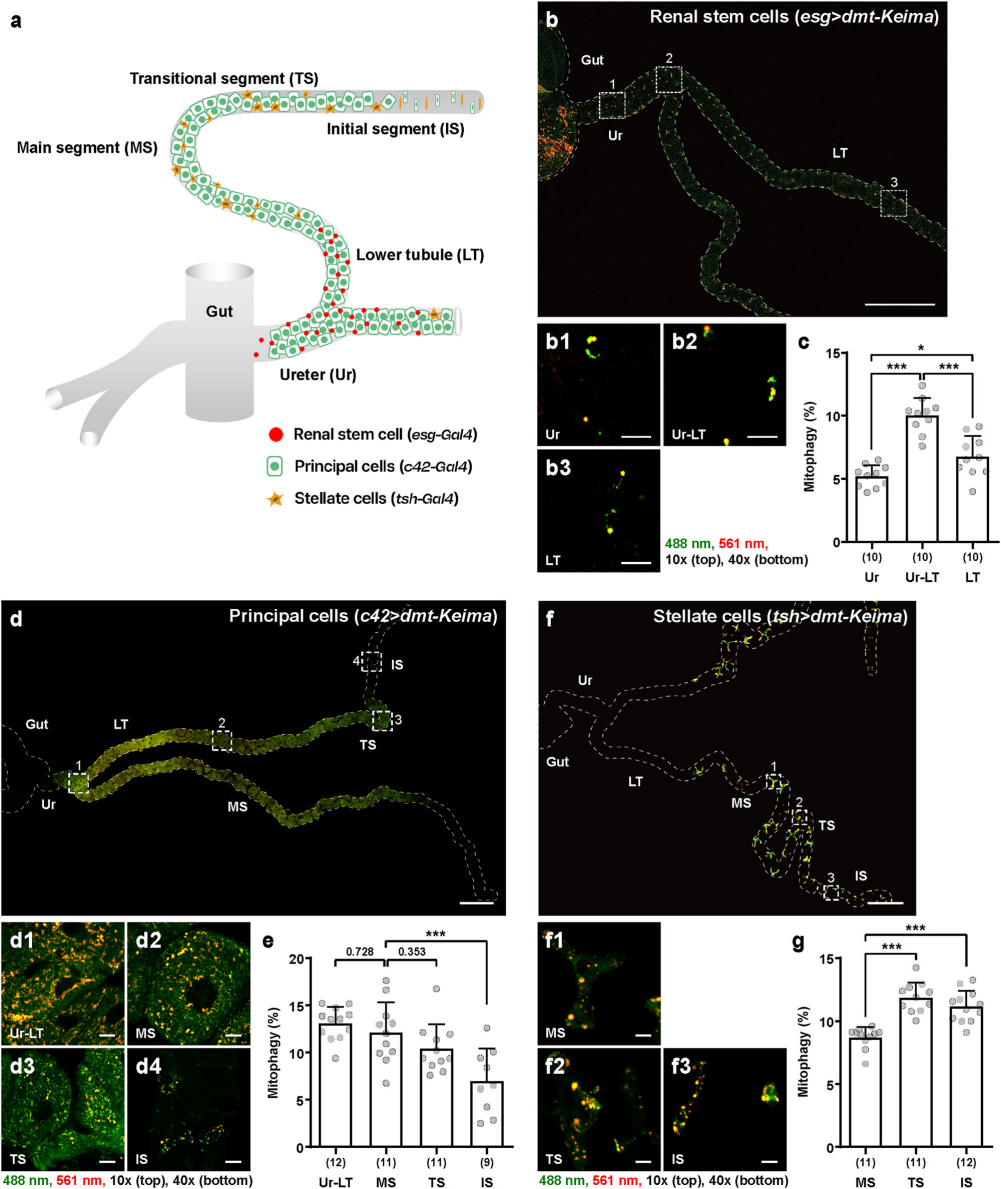
Mitophagy level depends on the cell type and structural location within the Malpighian tubules of *Drosophila.* **a** A structural diagram of the specific distributions of cell types in Malpighian tubules. Renal stem cells (*esg-GAL4*) can be found in the ureter (Ur) and lower tubule (LT). Principal cells (*c42-GAL4*) are distributed throughout the entire tubule, whereas stellate cells (*tsh-GAL4*) are located in the main segment (MS), transitional segment (TS) and initial segment (IS). **b**–**g** Confocal images of renal stem cells (**b**) principal cells (**d**) and stellate cells (**f**) in Malpighian tubules from male flies carrying *UAS-dmt-Keima* with cell type-specific drivers, with the quantitative analysis of mitophagy in the indicated regions of each cell type (**c**, **e** and **g** respectively). In **b**, **d** and **f** the boxed regions are presented in an enlarged format in the lower panel for a more detailed view. Scale bars, 200 μm (top) and 10 μm (bottom). In **c**, **e** and **g** the data are shown as mean ± s.d., and the mitophagy levels of *n* = 9–12 individual flies were quantified. dmt-Keima fluorescence was imaged at 488 nm (green) and 561 nm (red); mitophagy levels were quantified based on the red/green fluorescence ratio, which reflects mitochondrial acidification. The 10× and 40× indicate the magnifications of the objective lenses used for confocal imaging. The numbers in parentheses indicate *n*. Significance was determined by a one-way ANOVA with Šidák correction. The numbers above the bars are the *P* values. *P < 0.05 and ***P < 0.001.

To further investigate mitophagy in Malpighian tubules, we next analyzed the mitophagy levels of different cell types in *Drosophila* Malpighian tubules. Malpighian tubules consist of three major cell types, renal stem cells, principal cells and stellate cells^23^, which are specifically targeted by *esg-GAL4*, *c42-GAL4* and *tsh-GAL4* drivers, respectively (Fig. 2a). The cell type specificity of these drivers has been well validated in previous studies^34,35^. Consistent with this, dmt-Keima expression was restricted to cells in the expected regions, and their morphology, as visualized by confocal microscopy, clearly matched the characteristic features of each corresponding cell type (Fig. 2b, d, f). The secretory functions of Malpighian tubules in flies expressing dmt-Keima under the control of *esg-GAL4*, *c42-GAL4* and *tsh-*GAL4 were comparable to those in the control group (Supplementary Fig. 2a), suggesting that dmt-Keima expression in each cell type also did not interfere with Malpighian tubule function. The fidelity of the dmt-Keima system was also verified through NH_4_Cl treatment, *ATG5* knockdown and comparison with mitoQC upon cell type-specific expression of dmt-Keima (Supplementary Fig. 2b–e). As each type of cell has a different function and activity depending on its location in the Malpighian tubules^36,37^, we assessed the mitophagy of each cell type in several different locations in the Malpighian tubules. When we assessed the mitophagy levels of renal stem cells at three different regions from the ureter to the lower tubule, we found that mitophagy levels at the connection region between the ureter and the lower tubule (Fig. 2b(2), Ur–LT) were significantly higher than those at the ureter (Fig. 2b(1), Ur) and lower tubule regions (Fig. 2b(3), LT) (Fig. 2b, c). Principal cells at the connection region between the ureter and the lower tubule (Fig. 2d(1), Ur–LT) exhibited the highest mitophagy level compared with the main segment (Fig. 2d(2), MS) and transitional segment (Fig. 2d(3), TS) although this difference was not statistically significant (Fig. 2d(4), IS) (Fig. 2d, e). The principal cells in the initial segment exhibited much lower mitophagy (Fig. 2d, e). In contrast, stellate cells presented different patterns of mitophagy. The mitophagy levels of stellate cells in the main segment (Fig. 2f(1), MS) were lower than those of stellate cells in the transition segment (Fig. 2f(2), TS) and the initial segment (Fig. 2f(3), IS) (Fig. 2f, g). These results suggest that mitophagy may be closely related to the function of each cell type in Malpighian tubules.

### Mitophagy is essential for maintaining the secretory function of Malpighian tubules

**Fig. 3:**
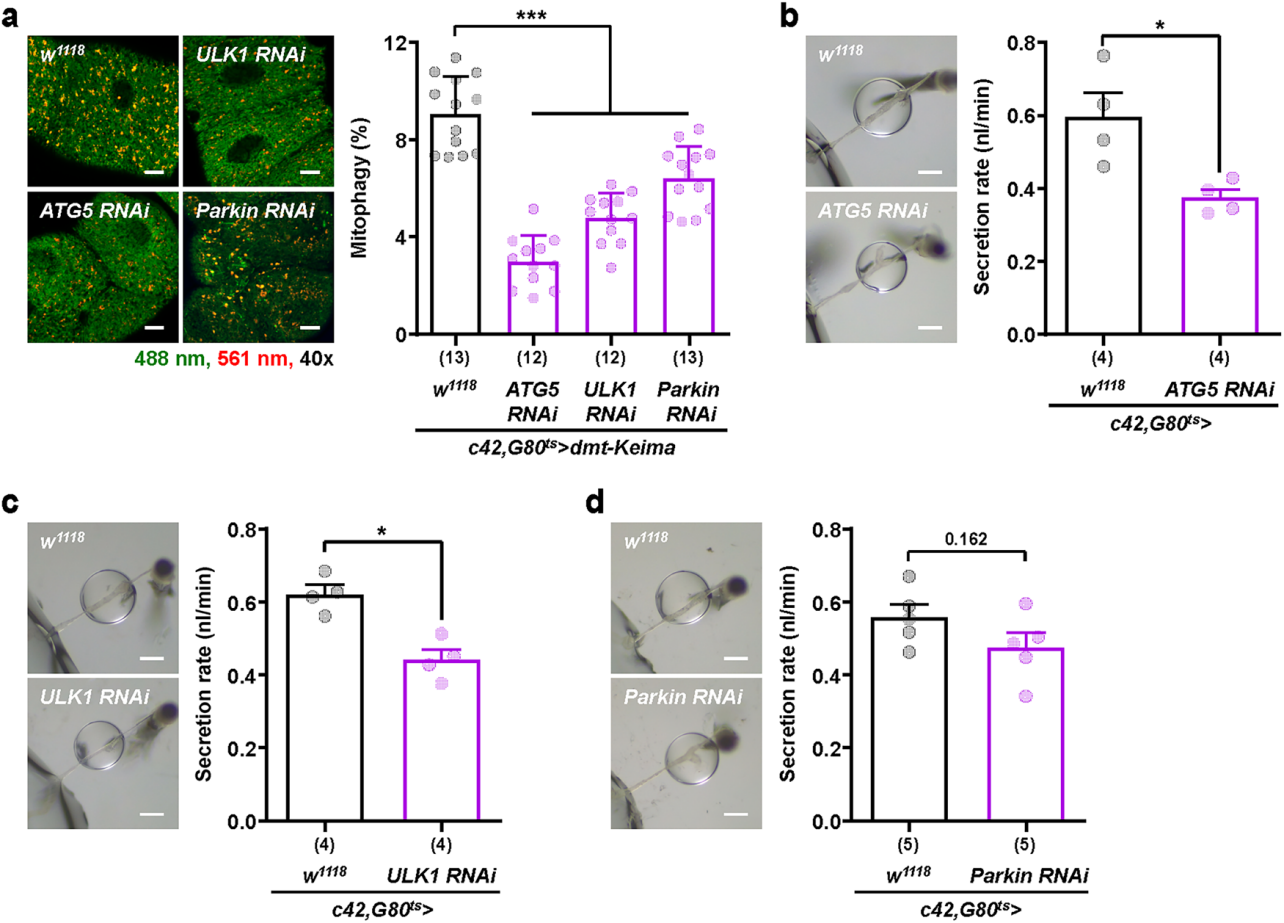
Reduction in mitophagy causes functional defects in Malpighian tubules. **a** Representative dmt-Keima fluorescence images (left) and quantitative mitophagy levels (right) of principal cells in the main segment from 7-day-old adult fly males under each RNAi after eclosion. Mitophagy was quantified by the red-to-green fluorescence ratio of dmt-Keima (561/488 nm), which indicates mitochondrial acidification. The data are shown as mean ± s.d. (*n* = 12–13). Scale bars, 10 μm. **b**–**d**. Representative images of fluid droplets from the Malpighian tubules of male flies of the genotypes *ATG5* RNAi (**b**), *ULK1* RNAi (**c**) and *Parkin* RNAi (**d**) (left), with the fluid secretion rates for Malpighian tubules from each fly strain (right). The data are shown as mean ± s.e.m. (4–5 biological replicates per group). Scale bars, 200 μm. The 40× indicates the magnification of the objective lens used for confocal imaging. The numbers in parentheses indicate *n*. Significance was determined by a one-way ANOVA with Šidák correction (**a**) or Student’s *t-*test (**b**–**d**). The numbers above the bars are the *P v*alues. *P < 0.05 and ***P < 0.001.

To investigate the relationship between mitophagy activity and cellular function in the Malpighian tubule, we next analyzed the impact of inhibiting mitophagy activity on secretion in principal cells, which are responsible for ATP-dependent active transport and secretion in the Malpighian tubule^38,39^. To inhibit mitophagy activity in principal cells, we knocked down several representative mitophagy genes, including *ATG5*, *ULK1* and *Parkin*, which are involved in autophagosome formation, mitophagy initiation and damaged mitochondria tagging^2,6^ (Supplementary Fig. 3a–c). To avoid possible side effects during development, we utilized the *c42, G80*^*ts*^ driver (*c42-Gal4, tubP-Gal80*^*ts*^)^40^ for adult-specific knockdown. Before performing these experiments, we confirmed that the mitophagy levels of dmt-Keima expressed on chromosome 2 (*UAS-dmt-Keima*/+; *da-Gal4*/+) and chromosome 3 (*da-Gal4*/*UAS-dmt-Keima*) were identical (Supplementary Fig. 3d). The knockdown of *ATG5* resulted in an approximately 65% reduction in the mitophagy level of principal cells (Fig. 3a). *ULK1* and *Parkin* knockdown resulted in milder reductions in mitophagy activity, with decreases of approximately 47% and 29%, respectively (Fig. 3a). Interestingly, the Ramsay assay results indicated that the knockdown of ATG5 led to the most notable decrease in secretory function (approximately 37%), whereas *ULK1* knockdown resulted in a reduction of approximately 29% (Fig. 3b, c). In contrast, Parkin knockdown induced an approximately 15% decrease in secretion, but this decrease was not statistically significant (Fig. 3d). These results suggest a direct relationship between mitophagy activity and secretion in the principal cells of Malpighian tubules.

### Decrease in mitophagy levels before functional defects in a *Drosophila* DKD model

**Fig. 4:**
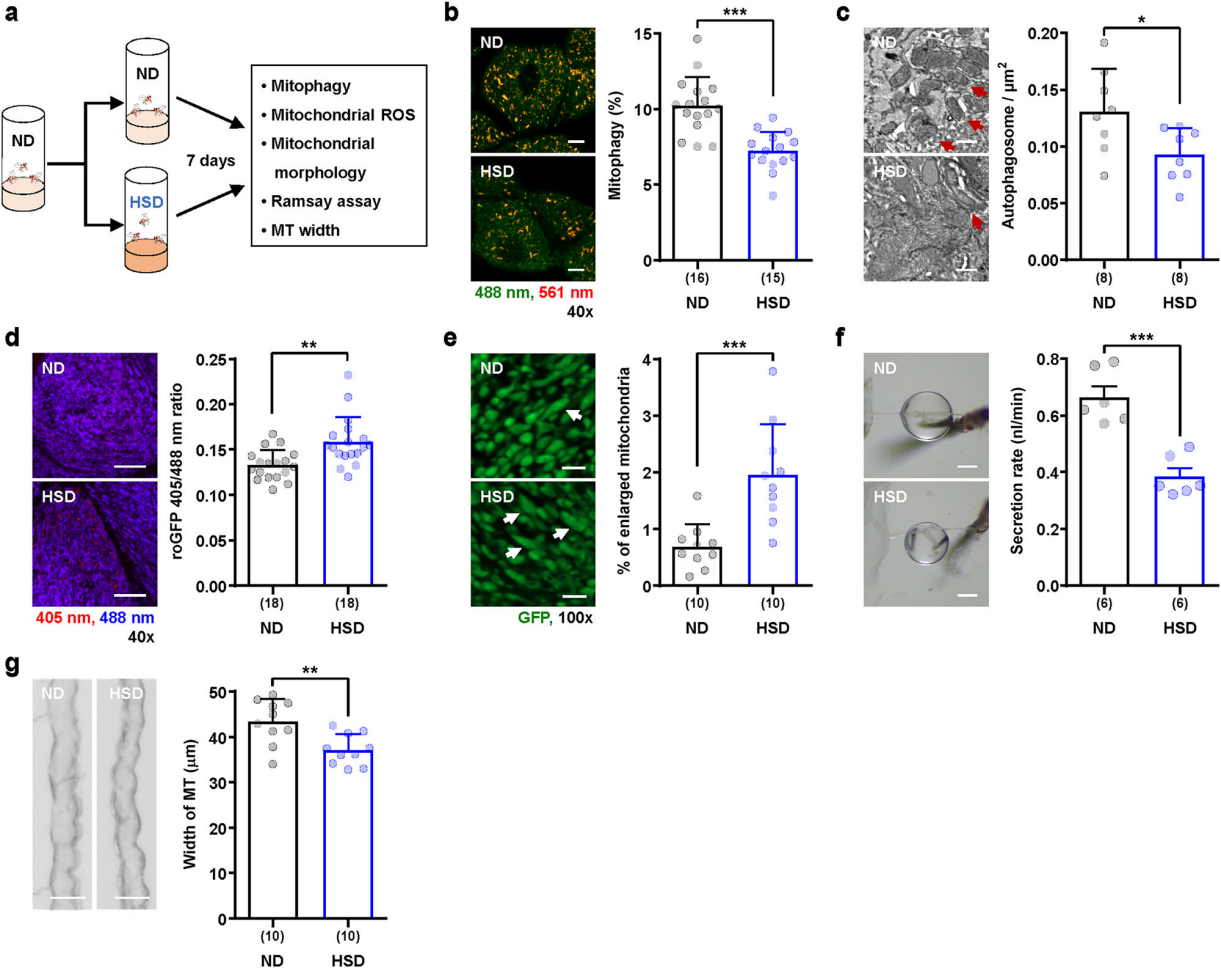
A HSD leads to mitochondrial impairments and functional anomalies in Malpighian tubules. **a** The experimental scheme for feeding a ND or HSD to establish DKD model. Newly eclosed male flies were fed a ND or HSD for seven consecutive days. **b** Representative dmt-Keima fluorescence images (left) and quantitative mitophagy levels (right) of principal cells in the main segment from 7-day-old adult male flies carrying *c42>dmt-Keima* under ND or HSD conditions. Mitophagy was assessed by the red-to-green fluorescence ratio of dmt-Keima (561/488 nm), which indicates mitochondrial acidification. The data are shown as mean ± s.d. (*n* = 15–16). Scale bars, 10 μm. **c** Representative scanning electron microscopy images (left) and the number of autophagosomes per 1 μm^2^ (right) in the main segment of *w*^*1118*^ male flies under each condition. The red arrows indicate autophagosomes. The data are shown as mean ± s.d. (*n* = 8). Scale bars, 0.5 μm**. d** Representative fluorescence images of mito-roGFP2-Orp1, a mitochondrial ROS reporter (left), and quantification of ROS levels measured via fluorescence intensity exchange (right) of principal cells in the main segment of male flies carrying *c42>mito-roGFP2-Orp1* under each condition. The data are shown as mean ± s.d. (*n* = 18). Scale bars, 10 μm. **e** Representative mitoHAGFP fluorescence images (left) and percentage of enlarged mitochondria (>2 μm^2^, right) of principal cells in the main segment of male flies carrying *c42>mitoHAGFP* under each condition. The white arrows indicate enlarged mitochondria. The data are shown as mean ± s.d. (*n* = 10). Scale bars, 2 μm. **f** Representative images of fluid droplets from the Malpighian tubules of male flies under each condition (left) and fluid secretion rates for Malpighian tubules from *w*^*1118*^ male flies (right). The data are shown as mean ±s.e.m. (6 biological replicates per group). Scale bars, 200 μm. **g** Representative images of the main segments in Malpighian tubules (left) and the measured width of the main segments (right) of *w*^*1118*^ male flies under each condition. The data are shown as mean ± s.d. (*n* = 10). Scale bars, 50 μm. The 40× and 100× indicate the magnifications of the objective lenses used for confocal imaging. The numbers in parentheses indicate *n*. Significance was determined by Student’s *t-*test. **P < 0.01 and ***P < 0.001.

Recent studies have suggested a link between mitophagy and renal diseases, including DKD^19^. However, direct analyses of mitophagy activity in models of renal disease have yet to be performed. To investigate the role of mitophagy in renal disease, we analyzed mitophagy activity in a DKD model in *Drosophila*. Previous studies have shown that the continuous consumption of a 1 M sucrose high-sugar diet (HSD) recapitulates type 2 diabetes and the DKD phenotype in *Drosophila*^41–43^. Consistent with previous reports^41–43^, we observed increased uric acid accumulation and kidney stone formation following HSD treatment, confirming that our protocol effectively induced DKD phenotypes in *Drosophila* (Supplementary Fig. 4a–c). To investigate the alterations in mitophagy activity associated with DKD development, we analyzed mitophagy levels specifically in the principal cells of *c42>dmt-Keima* flies after 1 week of HSD feeding (Fig. 4a). Interestingly, mitophagy levels in principal cells decreased by approximately 29% after 1 week of HSD feeding (Fig. 4b). This reduction was further validated via the use of an independent mitophagy reporter, mitoQC, which resulted in a similar decrease in mitophagy activity following HSD treatment (Supplementary Fig. 4d). Moreover, electron microscopy analysis revealed a marked reduction in the number of autophagosomes in the principal cells of HSD-fed flies (Fig. 4c). Consistently, the fluorescence signal of mitochondrial marker protein mitoHAGFP increased significantly under HSD feeding conditions (Supplementary Fig. 4e). This decrease in mitophagy was accompanied by a significant increase in mitochondrial reactive oxygen species (ROS) levels and the proportion of enlarged mitochondria (Fig. 4d, e), a characteristc of dysfunctional mitochondria^44,45^, suggesting that mitochondrial dysfunction had developed upon HSD feeding. In addition, HSD feeding also resulted in a significant decrease in the secretion rate and width of Malpighian tubules (decreases of 42% and 14%, respectively) (Fig. 4f, g), confirming that the DKD phenotypes were well developed, which is consistent with previous reports^41,43^. These results revealed a significant decrease in mitophagy activity in the Malpighian tubules of the *Drosophila* DKD model.

**Fig. 5:**
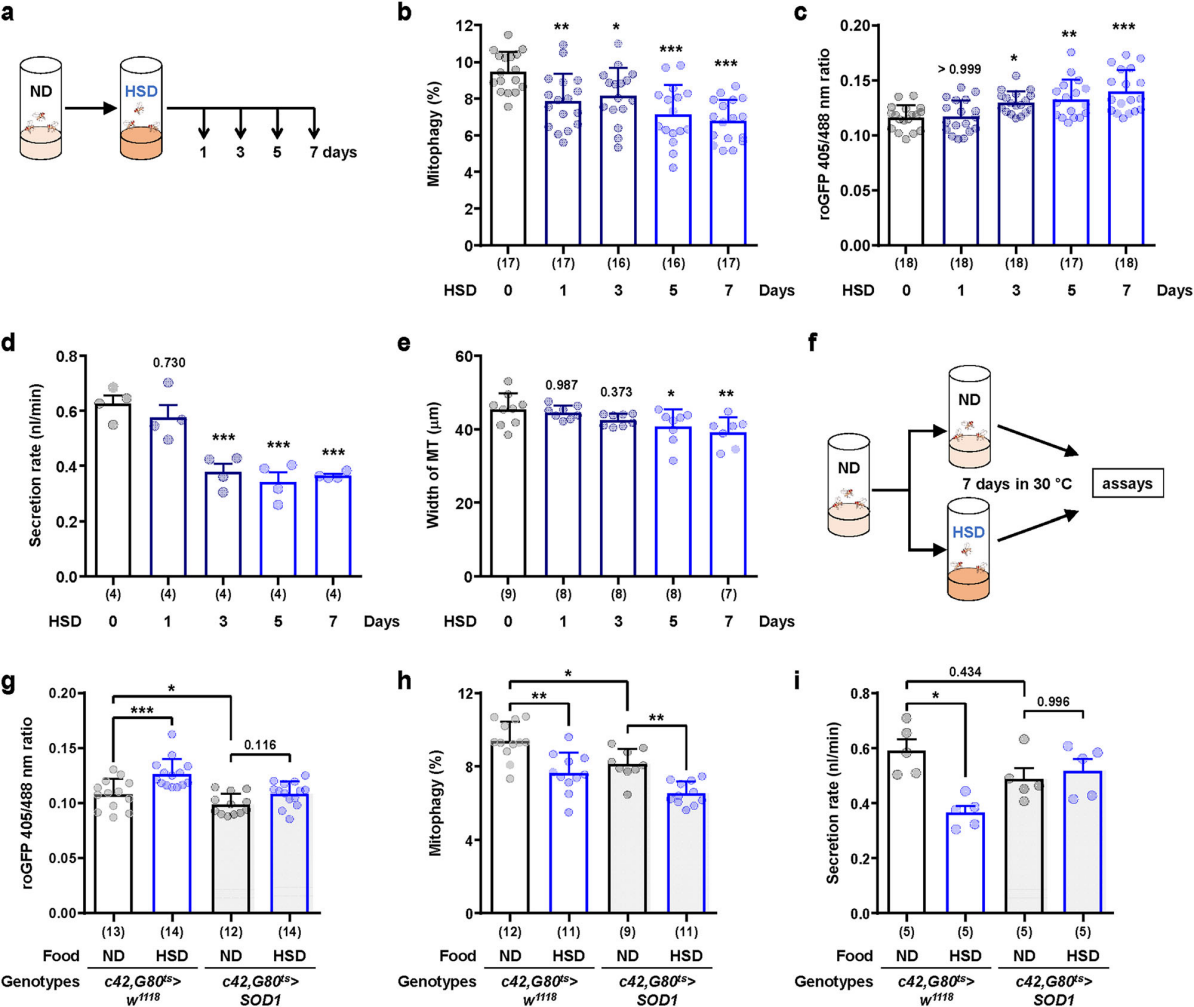
HSD induces a decrease in mitophagy in Malpighian tubules, followed by subsequent functional abnormalities. **a** The experimental design for feeding a ND or HSD (for the results shown in **b**–**e**). Newly eclosed male flies were administered a HSD for the indicated periods. The control group was maintained on a ND for 7 days. Each HSD group was provided with a HSD on the final day, for the last 3 days, for the last 5 days or for all 7 days. **b**, **c** Quantitative mitophagy level (**b**) or ROS level (**c**) of principal cells in the main segment from 7-day-old adult male flies fed a HSD for 1–7 days. The data are shown as mean ± SDs (*n* = 16-18). **d** Fluid secretion rate of Malpighian tubules from male flies under each condition. The data are shown as mean ± s.e.m. (4 biological replicates per group). **e** Measured width of the main segments from male flies under each condition. The data are shown as mean ± s.d. (*n* = 7–9). **f** The experimental design for feeding a ND or HSD to adult flies carrying *c42-Gal4*, *tub-Gal80*^*ts*^ (for the results shown in **g**–**i**). The flies were maintained for 7 days in 30 °C for SOD1 expression. **g**, **i** Quantitative ROS (**g**) and mitophagy (**i**) levels in principal cells from control or SOD1 overexpressing flies. The data are shown as mean ± s.d. (*n* = 9–14). **h** Fluid secretion rate of Malpighian tubules from male flies under the indicated genotypes and conditions. The data are shown as mean ± s.e.m. (5 biological replicates per group). The numbers in parentheses indicate *n*. Significance was determined by a one-way ANOVA (**b**–**e**) with Šidák correction compared with the control group or two-way ANOVA (**g**–**i**) with Šidák correction. The numbers above the bars are the *P v*alues. *P < 0.05, **P < 0.01 and ***P < 0.001.

To understand the role of reduced mitophagy activity in the development of DKD phenotypes, we conducted a time-course analysis of mitophagy activity, mitochondrial dysfunction and secretion upon HSD feeding (Fig. 5a). Interestingly, a decrease in mitophagy activity was observed earlier than an increase in mitochondrial ROS and a decrease in secretory function. Mitophagy activity decreased by approximately 17% as early as 1 day after HSD feeding, with a further reduction of approximately 25% observed at 5 days after HSD feeding (Fig. 5b). In contrast, an increase in mitochondrial ROS levels was observed beginning at the 3-day time point after HSD feeding (Fig. 5c). Similarly, decreases in the secretion rate and width of Malpighian tubules were observed at 3 days and 5 days, respectively, after HSD feeding (Fig. 5d, e). These results suggest that impaired mitophagy activity may play a causative role in inducing mitochondrial dysfunction and functional defects in Malpighian tubules during the development of DKD.

To test whether ROS mediate mitophagy suppression, we overexpressed SOD1 via the *c42, G80*^*ts*^ driver (Fig. 5f and Supplementary Fig. 5c). While SOD1 blocked the HSD-induced increase in mitochondrial ROS and restored fluid secretion (Fig. 5g, i), it did not restore mitophagy activity (Fig. 5h), suggesting that ROS are not the primary cause of HSD-induced mitophagy reduction. Similarly, the overexpression of AMPKα or *Tor* knockdown, which are known mitophagy activators^2,28^, failed to prevent the decrease in mitophagy caused by HSD (Supplementary Fig. 5a, b, d, e). These results indicate that HSD suppresses mitophagy through mechanisms independent of ROS, AMPK or mTOR signaling (Fig. 5f–i and Supplementary Fig. 5a–e).

### Mitophagy stimulation rescues mitochondrial dysfunction and impaired secretion in the Malpighian tubules of the *Drosophila* DKD model

**Fig. 6:**
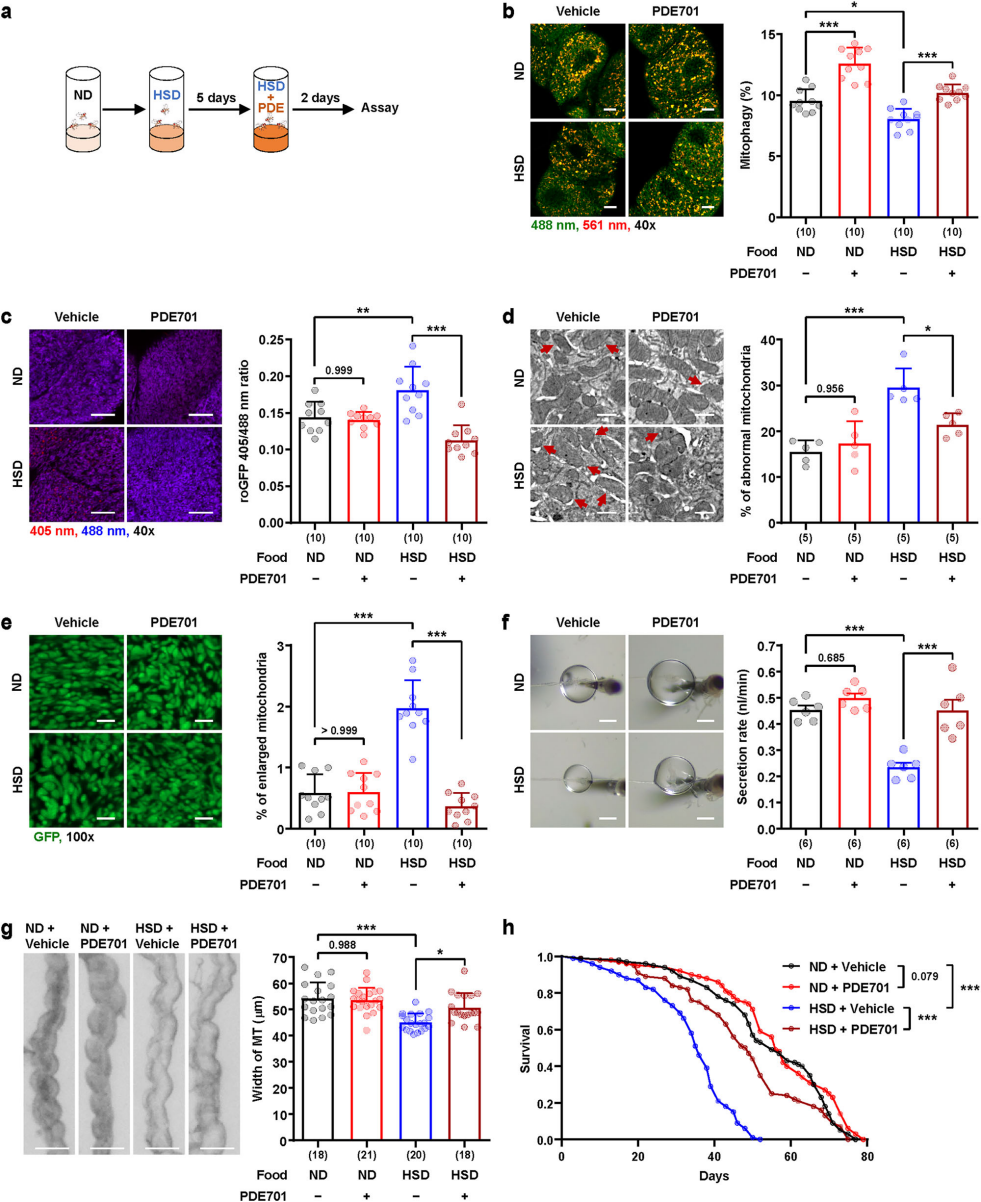
Mitophagy induction rescues HSD-induced Malpighian tubule dysfunction. **a** The experimental design for feeding a HSD and administering the mitophagy inducer PDE701 to adult flies. Naïve male flies were maintained on a ND or HSD for 5 days and then transferred to food supplemented with PDE701 (200 μm) for 2 days before the subsequent assays. **b** Representative dmt-Keima fluorescence images (left) and quantitative mitophagy levels (right) of principal cells in the main segment from male flies carrying *c42>dmt-Keima* fed a ND or HSD with or without PDE701 treatment. Mitophagy was assessed by the red-to-green fluorescence ratio of dmt-Keima (561/488 nm), which indicates mitochondrial acidification. The data are shown as mean ± s.d. (*n* = 10). Scale bars, 10 μm. **c** Representative mito-roGFP2-Orp1 fluorescence images (left) and quantitative ROS levels (right) of principal cells in the main segment of male flies carrying *c42>mito-roGFP2-Orp1* under the indicated conditions. The data are shown as mean ± s.d. (*n* = 10). Scale bars, 10 μm. **d** Representative scanning electron microscopy images and the proportion of abnormal mitochondria (right) in the main segment of male flies under each condition (left). The red arrows indicate abnormal mitochondria with disrupted cristae structures. The data are shown as mean ± s.d. (*n* = 5). Scale bars, 0.5 μm. **e** Representative mitoHAGFP fluorescence images (left) and the percentage of enlarged mitochondria (>2 μm^2^, right) of principal cells in the main segment from male flies carrying *c42>mitoHAGFP* under the indicated conditions. The data are shown as mean ± s.d. (*n* = 10). Scale bars, 2 μm. **f** Representative images of fluid droplets (left) and the measured secretion rates (right) of Malpighian tubules from *w*^*1118*^ male flies under the indicated conditions. The data are shown as mean ± s.e.m. (6 biological replicates per group). Scale bars, 200 μm. **g** Representative images of the main segments of Malpighian tubules (left) and the measured width of the main segments (right) from *w*^*1118*^ male flies under the indicated conditions. The data are shown as mean ± s.d. (*n* = 18–21). Scale bars, 50 μm. **h** A survival curve for the lifespan of *n* = 100 *w*^*1118*^ male flies under the indicated conditions. The numbers in parentheses indicate *n*. Significance was determined by a two-way ANOVA with Šidák correction (**b**–**f**) or the log-rank test (**g**). The numbers above the bars are the *P v*alues. *P< 0.05, **P < 0.01 and ***P < 0.001.

The stimulation of mitophagy has emerged as a promising strategy for the treatment of various human diseases^7,46^. To verify the role of mitophagy reduction during DKD development and assess the therapeutic effect of mitophagy stimulation, we employed a novel mitophagy-specific inducer PDE701, which was recently identified by our group^47^. We observed that feeding with PDE701 (200 μM) significantly increased mitophagy activity in Malpighian tubules by approximately 20%, as measured by both dmt-Keima and mitoQC reporters (Supplementary Fig. 6a, b). This increase was further supported by a reduction in the mitochondrial protein SDHB detected by western blotting and a decrease in mitochondrial abundance assessed by mitoHAGFP (Supplementary Fig. 6c, d). To investigate whether mitophagy induction mitigates defects induced by HSD feeding, we first fed *Drosophila* a HSD for 5 days and then treated them with the mitophagy inducer PDE701 (200 μM) for an additional 2 days (Fig. 6a). Treatment with PDE701 for 2 days successfully reversed the HSD-induced reduction in mitophagy levels to the level observed in the normal diet (ND) group (Fig. 6b). Importantly, PDE701 treatment also rescued the increased mitochondrial ROS level induced by HSD feeding (Fig. 6c). The increased percentage of abnormal mitochondria observed by electron microscopy, as well as enlarged mitochondria assessed by fluorescence imaging—both indicators of mitochondrial dysfunction upon HSD feeding—were also reduced by PDE701 treatment (Fig. 6d, e), suggesting that the mitochondrial dysfunction induced by HSD feeding was ameliorated upon PDE701 treatment. Moreover, the decreased secretion ability and width of Malpighian tubules were also rescued upon PDE701 treatment (Fig. 6f, g). In addition, the decrease in lifespan caused by HSD feeding was reversed significantly by cotreatment with PDE701 (Fig. 6h and Supplementary Fig. 6e). The median lifespan was decreased by approximately 35% by HSD feeding (from 55 days to 36 days), and PDE701 cotreatment resulted in a recovery of approximately 49% (from 36 days to 49 days). These results suggest that the functional defects and mitochondrial dysfunction induced by HSD in Malpighian tubules were reversed by PDE701 treatment. Compared with rotenone, a well-known mitophagy inducer^2,4^, PDE701 achieved similar levels of mitophagy activation at a much lower concentration and exhibited markedly lower toxicity in a survival assay (Supplementary Fig. 6f–h), highlighting its favorable therapeutic potential.

**Fig. 7:**
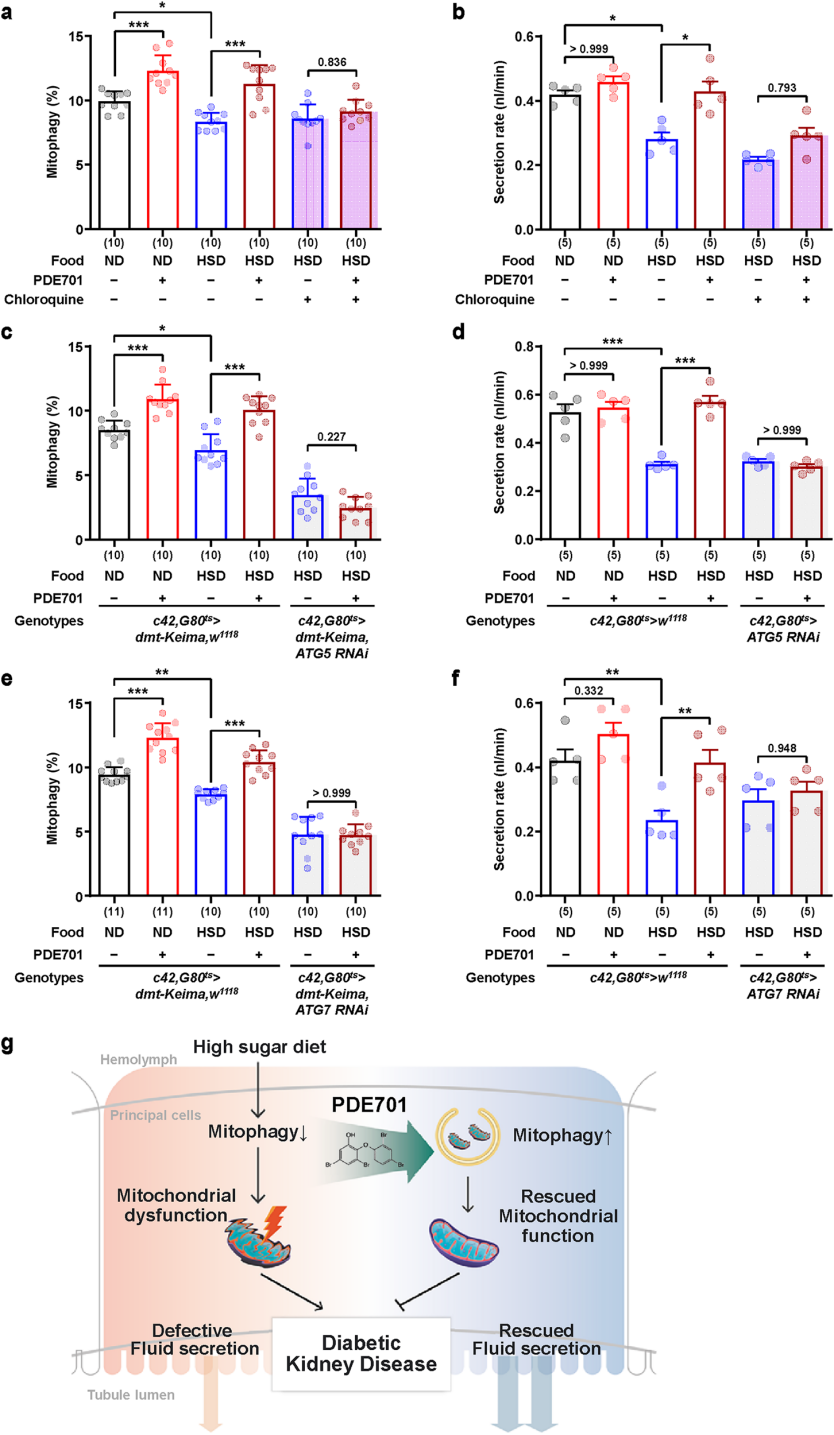
PDE701 rescues DKD through mitophagy induction. **a** Quantification of mitophagy in principal cells in the main segment from male flies carrying *c42>dmt-Keima* reared on a ND or HSD and treated with PDE701 and chloroquine, following the experimental design shown in Fig. 6a. The data are shown as mean ± s.d. (*n* = 10). **b** Fluid secretion rate of Malpighian tubules from *w*^*1118*^ male flies under each condition. The data are shown as mean ± s.e.m. (5 biological replicates per group). **c**–**f** Quantification of mitophagy in principal cells in the main segment from control, *ATG5* RNAi (**c**) or *ATG7* RNAi (**e**) male fli**e**s reared on a ND or HSD and treated with PDE701 and the fluid secretion rates of Malpighian tubules from control, *ATG5* RNAi (**d**) or *ATG7* RNAi (**f**) male flies under each condition. The data in **c** and **e** are shown as mean ± s.d. (*n* = 10–11). The data in **d** and **f** are shown as mean ± s.e.m. (5 biological replicates per group). The numbers in parentheses indicate *n*. Significance was determined by a two-way ANOVA with Šidák correction. The numbers above the bars are the *P v*alues. * *P* < 0.05, **P < 0.01 and ***P < 0.001. **g** A graphical summary of the effects of PDE701 treatment on DKD. HSD feeding reduced mitophagy and promoted mitochondrial dysfunction in Malpighian tubules. PDE701 feeding induced mitophagy and reversed HSD-induced defects in Malpighian tubules.

To verify whether the therapeutic effect of PDE701 on the DKD model is dependent on mitophagy, we tested the effect of chloroquine, an inhibitor of autophagosome‒lysosome fusion, which blocks the final degradation step of mitophagy. Chloroquine treatment abolished the improvements in mitophagy and secretion rates mediated by PDE701 during HSD feeding (Fig. 7a, b). Furthermore, the PDE701-mediated alleviation of mitophagy reduction and secretion was also abolished by the knockdown of *ATG5* or *ATG7* (Fig. 7c–f and Supplementary Figs. 3a and 7c), which is consistent with our previous finding that PDE701-mediated therapeutic effect is suppressed by both *ATG5* and *ATG7* knockdown^47^. These results suggest that PDE701 ameliorated HSD-induced Malpighian tubule dysfunction through mitophagy stimulation (Fig. 7g). In addition, knockdown of *Parkin* or *AMPKα* did not block PDE701-induced mitophagy (Supplementary Fig. 7a, b, d), suggesting that PDE701 acts via a distinct pathway from these canonical regulators in both mammalian cells^47^ and *Drosophila* principal cells. Morever, although PDE701-mediated mitophagy requires ATG5 and ATG7, PDE701 treatment did not affect their mRNA or protein expression in both *Drosophila* and human cells (Supplementary Fig. 8a–e), suggesting that PDE701 promotes mitophagy through mechanisms other than direct regulation of these genes.

## Discussion

Despite recent attention to the role of mitophagy in kidney function and pathogenesis^11–14^, the precise analysis of kidney mitophagy remains largely undetermined. In this study, we developed an advanced transgenic *Drosophila* model, dmt-Keima, that allows direct measurement of mitophagy in Malpighian tubules, an analog of the kidney in *Drosophila*. Our analysis of mitophagy activity in dmt-Keima *Drosophila* revealed that Malpighian tubules have stronger mitophagy activity than other tissues, such as the intestine and muscle. Similarly, McWilliams et al. reported a high level of mitophagy activity in mouse kidney tissue compared with other organs^33^. Elevated mitophagy in kidney tissue suggests that mitophagy plays a crucial role in maintaining kidney function, as the kidney is vulnerable to mitochondrial damage because of its involvement in various metabolic processes and detoxification^15–17^. Indeed, our results revealed that the inhibition of mitophagy through the knockdown of mitophagy genes resulted in a reduction in the secretory function of Malpighian tubules. Whereas ATG5 or ULK1 knockdown led to a significant decrease in mitophagy activity and secretion, knockdown of the Parkin gene had little effect on either mitophagy activity and secretion. These results are consistent with those of prior studies by our group and others that demonstrated that, in contrast to its role in stress-induced mitophagy, the PINK1–Parkin pathway has a limited role in basal mitophagy^22,48–51^, implying that it is not a key factor in maintaining basal mitophagy activity in the kidney.

Importantly, our study provides evidence supporting the notion that the decrease in mitophagy activity induced by HSD contributes to the development of DKD phenotypes in Malpighian tubules. Previous studies have suggested an association between impaired mitophagy and kidney diseases, including DKD^11–14^. For example, decreased mitophagy markers, such as PINK1, Parkin, LC3II, Mfn1 and Beclin1, have been observed in different renal cell types, tubular epithelial cells, podocytes and glomerular mesenchymal cells in in vitro/in vivo DKD models^52–55^. Consistent with these studies, we revealed that mitophagy activity in principal cells significantly decreased in response to HSD, as observed via the dmt-Keima system. Notably, this decline in mitophagy activity in principal cells occurred earlier than the onset of mitochondrial dysfunction or functional defects in Malpighian tubules did, suggesting that impaired mitophagy may play a causative role in DKD pathogenesis. Recent studies have also shown that modulating mitophagy affects the onset of DKD through mechanisms such as inflammation inhibition and renal cell death prevention^52,56–58^. Consistently, studies in mouse models have demonstrated that reduced function of ULK1, ATG5 or ATG7 in kidney cells is closely associated with various kidney diseases, including DKD^59,60^. In this context, we showed that the mitophagy inducer PDE701 ameliorated mitochondrial dysfunction and alleviated functional and structural abnormalities in the kidneys, underscoring the therapeutic potential of mitophagy inducers in DKD. To advance its therapeutic application, we are currently performing affinity purification experiments to identify the direct binding partners of PDE701, which we expect to provide important insights into the molecular mechanisms underlying PDE701-induced mitophagy.

Importantly, there are physiological differences between the Malpighian tubules in *Drosophila* and the human kidney. While the human kidney utilizes a filtration-based mechanism through the glomerulus, Malpighian tubules function primarily through secretion-driven processes to regulate ionic balance and waste excretion^61,62^. Despite these differences, conserved cellular and molecular pathways, such as mitochondrial dynamics and mitophagy, allow Malpighian tubules to serve as valuable models for investigating kidney-related pathologies, including DKD^23–25^. Therefore, validation of our results in mammalian models is essential to further confirm their relevance and enhance their translational potential. Future studies will be conducted to validate these findings in mouse models and human kidney cells and to confirm their physiological and clinical relevance. Our study also raises the question of how HSD leads to a reduction in mitophagy activity. Previous studies have reported that high glucose or hyperglycemic conditions induce mitochondrial dysfunction through elevated ROS levels and impaired control of mitochondrial dynamics^63,64^. It is possible that HSD reduces mitophagy in Malpighian tubules through similar mechanisms. Determining the exact pathway by which HSD reduces mitophagy could provide valuable insights into the molecular mechanisms underlying DKD pathogenesis.

Notably, the levels of mitophagy in the major cell types of Malpighian tubules—renal stem cells, principal cells and stellate cells—vary depending on their location within the tubules. We have previously shown, through studies of mouse and *Drosophila* tissues, that mitophagy activity is highly sensitive to the functional state of cells and their environmental context^21,22^. Thus, our results suggest that the function and microenvironment of these cells may differ according to their location in Malpighian tubules. Indeed, it has been recently reported that renal stem cells begin their differentiation near the ureter and progress toward the lower tubule, eventually differentiating into principal cells^35^. Additionally, principal cells are primarily active in the main segment, whereas stellate cells function mainly in the initial segment^36,37^. Thus, the differences in mitophagy activity across locations within Malpighian tubules could be attributed to the functional characteristics and energy demands of each cell type. In this study, we verified that mitophagy is essential for the secretory function of principal cells; whether mitophagy is similarly necessary for the functions of renal stem cells and stellate cells remains an interesting topic for future research. Although the mt-Keima-based mitophagy assay is more sensitive than other currently available methods are^65^, further validation with alternative methods would be beneficial.

Taken together, our results reveal that renal mitophagy is crucial for maintaining mitochondrial homeostasis and kidney function. Furthermore, we revealed that reduced mitophagy activity in DKD models and enhanced mitophagy can have therapeutic effects on DKD. While the detailed molecular mechanism by which HSD reduces mitophagy in the kidney needs to be further investigated, the results of our study suggest that mitophagy stimulation could be a promising strategy for preserving kidney function and treating DKD.

## Supplementary information

Supplementary Information
