# Supplementary material for: Exploring mitophagy levels in Drosophila Malpighian tubules unveils the pivotal role of mitophagy in kidney function and diabetic kidney disease
Source: Exp Mol Med. 2025 Oct 23;57(10):2364–75. doi: 10.1038/s12276-025-01558-2 (PMC12586521; doi:10.1038/s12276-025-01558-2)
Supplement: Supplementary file 1 — Supplementary Information [file 12276_2025_1558_MOESM1_ESM.pdf]

Supplementary Information

# Exploring mitophagy levels in *Drosophila* malpighian tubules unveils the pivotal role of mitophagy in kidney function and diabetic kidney disease

Kang-Min Lee[1,2,3], Jihun Kim[1,2,3], Hye Lim Jung[1,2,3], Young Yeon Kim[1,2,3], Jihoon Lee[4], Yeon-Ju Lee[4], Eunhee Yoo[5], Hyi-Seung Lee[4,*], and Jeanho Yun[1,2,3*]

[1]Department of Biochemistry, College of Medicine, Dong-A University, Busan, 49201, Republic of Korea

[2]Department of Translational Biomedical Sciences, Graduate School of Dong-A University, Busan, 49201, Republic of Korea

[3]Peripheral Neuropathy Research Center, Dong-A University, Busan, 49201, Republic of Korea.

[4]Korea Institute of Ocean Science & Technology (KIOST), Busan, 49111, Republic of Korea

[5]Altmedical Co., Ltd. Seoul, 02792, Republic of Korea

These authors contributed equally: Kang-Min Lee, Jihun Kim, Hye Lim Jung

* email: hslee@kiost.ac.kr; yunj@dau.ac.kr

## Methods

### Reagents and treatments

To establish a DKD model in *Drosophila*, a high sugar diet (HSD) was prepared by adding sucrose to freshly made standard fly media (normal diet, ND) to a final concentration of 1 M, as previously described[1]. HSD was administered to newly eclosed male flies for 7 consecutive days. PDE701 was isolated from the marine sponge *Dysidea* sp. through MeOH and $CH_2Cl_2$ extraction, followed by serial partitioning and reversed-phase chromatography. The active fractions were further purified via high-performance liquid chromatography (HPLC) with a YMC-ODS column, as previously described[2]. The purity of PDE701 (>99%) was confirmed by HPLC. For the administration of PDE701, rotenone (R8875, Sigma-Aldrich, St. Louis, MO, USA), or chloroquine (CQ; C6628, Sigma–Aldrich, St. Louis, MO, USA), the flies were transferred to fresh fly media supplemented with 200 μM PDE701, 5 mM rotenone, 200 μM CQ, or 0.2% DMSO as the vehicle. This treatment was maintained for 2 days prior to subsequent assays.

BEAS-2B (CRL-9609; ATCC, Rockville, USA) cells were maintained in DMEM containing 10% fetal bovine serum. For PDE701 treatment, 5 x $10^5$ cells were exposed to 6 μM PDE701 for 24 h.

### Ramsay assay

Fluid secretion rates of Malpighian tubules were measured via the Ramsay assay, which quantifies the volume of secreted fluid over time, as previously described[3]. For the Ramsay assay, 7-day-old naïve male flies were anesthetized on ice and dissected under *Drosophila* saline. The dissected anterior Malpighian tubules were moved to standard bathing medium (SBM). To secrete excretion fluid into the ureter, one tubule was placed in SBM, and the other

tubule was attached to a metal pin. Images of the fluid droplet secreted from the ureter were captured with a Leica EZ4E microscope (Leica, Wetzlar, Germany) after a 30-min interval, and ImageJ software (NIH, Bethesda, MD, USA) was used to calculate the volume ($V = 4/3\pi r^3$) and the secretion rate (volume/min). Fluid secretion was examined in 6-8 flies per group, and samples without any fluid droplets were excluded from the results. The experiments were repeated at least 4 times for quantification, and the results are presented as the mean values with the standard errors of the means (S.E.M.).

**Dissection and confocal imaging conditions for fluorescence analysis**

To perform fluorescence-based analysis via mt-Keima (mitophagy), mitoQC (mitophagy), mito-roGFP2-orp1 (mitochondrial ROS levels), and mitoHAGFP (mitochondrial mass and morphology), 7-day-old naïve male flies were anesthetized on ice and dissected in PBS (pH 7.4). Dissected tissues were placed in PBS on a glass slide and imaged via a Zeiss LSM 800 confocal microscope (Carl Zeiss, GmbH, Germany) equipped with Apochromat 10x/0.45 M27, c-Apochromat 40x/1.2 W Corr M27, and Apochromat 100x/1.4 M27 lenses at the Neuroscience Translational Research Solution Center (Busan, Republic of Korea). These dissection and imaging procedures were consistently applied across all fluorescence signal analyses described below. For analysis of the main segment of the Malpighian tubules, the position of #2 in Figure 2d of both anterior tubules per fly was imaged.

**Mitophagy quantification in Malpighian tubules**

Confocal images for mt-Keima fluorescence were acquired via the procedures described in the “Dissection and confocal imaging conditions for fluorescence analysis” section. dmt-

Keima fluorescence was imaged with two sequential excitation lasers (488 and 561 nm) using a 595-700 nm emission bandwidth. The mt-Keima fluorescence signals from the 488 and 561 nm excitation wavelengths are represented in green and red, respectively. The quantification of mitophagy based on the mt-Keima confocal images was performed using the Zeiss Zen software (Carl Zeiss, GmbH, Germany) on a pixel-by-pixel basis, as previously described[4,5]. Briefly, every pixel in the confocal image was plotted in the scatter diagram in the colocalization menu. The green fluorescence intensity (488 nm) is shown on the x-axis, and the red fluorescence intensity (561 nm) is shown on the y-axis. Pixels with a high red/green ratio are designated in the scatter diagram via crosshairs and quadrants. The mitophagy level (% of mitophagy) is defined as the number of pixels that have a high red/green ratio divided by the total number of pixels.

Confocal images of mitoQC fluorescence were acquired as described above. mitoQC fluorescence was imaged with two sequential excitation lasers (488 and 555 nm) using a 580 nm emission bandwidth. The quantification of mitophagy based on mitoQC confocal images was performed using the Zeiss Zen software (Carl Zeiss) on a pixel-by-pixel basis, similar to the quantification method used for mt-Keima confocal images. More than 7 fly samples were used for quantification, and the mean values were calculated. The results are presented as the mean values with the SDs.

**Measurement of mitochondrial ROS in Malpighian tubules**

To analyze mitochondrial ROS levels, confocal images of mito-roGFP2-orp1 fluorescence were acquired via the procedures described in the "Dissection and confocal imaging conditions for fluorescence analysis" section. mito-roGFP2-orp1 fluorescence was imaged with two sequential excitation lasers (405 and 488 nm) using a 500-530 nm emission bandwidth. The mito-roGFP2-orp1 fluorescence signals from the 405 and 488 nm excitation wavelengths are

represented in red and blue, respectively. The quantification of ROS based on the confocal images was performed using ImageJ software. A ratio image was generated by dividing the 405 nm image by the 488 nm image on a pixel-by-pixel basis with the "Ratio Plus" plugin. The 405/488 nm ratio reflects the oxidation state of mito-roGFP2-Orp1, a redox-sensitive GFP variant whose excitation properties shift in response to mitochondrial hydrogen peroxide ($H_2O_2$). An increased 405/488 ratio indicates a more oxidized mitochondrial environment and elevated ROS levels[6]. More than 10 fly samples were used for quantification, and the mean values were calculated. The results are presented as the mean values with the SDs.

**Measurement of mitochondrial levels in Malpighian tubules**

To analyze the level of mitochondria under each condition, confocal images of mitoHAGFP fluorescence were acquired via the procedures described in the "Dissection and confocal imaging conditions for fluorescence analysis" section. mitoHAGFP, a genetically encoded fluorescent protein that constitutively labels mitochondria, was imaged using a 488 nm excitation laser and a 410-520 nm emission bandwidth. The fluorescence intensity of mitoHAGFP, which correlates with mitochondrial content, was quantified to determine mitochondrial levels. More than ten fly samples were analyzed for each condition. The results are presented as the mean values with the SDs.

**Analysis of mitochondrial morphology in principle cells**

To examine the increase in dysfunctional mitochondria, confocal images of mitoHAGFP fluorescence were acquired via the procedures described in the "Dissection and confocal imaging conditions for fluorescence analysis" section. mitoHAGFP fluorescence was imaged with a 488 nm excitation laser and a 410-520 nm emission bandwidth. Mitochondrial morphology was analyzed as previously described[7]. Briefly, images were processed and

analyzed using ImageJ software. The total number of mitochondria and the number of enlarged mitochondria (defined as those larger than 2 $\mu m^2$) were quantified using the "Analyze Particles" tool. The percentage of enlarged mitochondria was then calculated for each sample. Ten fly samples were analyzed per group. The results are presented as the mean values with the SDs.

**Electron microscopy analysis**

For electron microscopy analysis, male flies were anesthetized on ice, and the main segment of the Malpighian tubules was dissected in PBS. The dissected tubules were fixed in 4% paraformaldehyde (P6148, Sigma-Aldrich) and 3% glutaraldehyde (G5882, Sigma-Aldrich) overnight. The samples were transferred to 0.05 M sodium cacodylate buffer (C0250, Sigma-Aldrich) and postfixed in 2% osmium tetroxide (#18463, $OsO_4$ ; Ted Pella, Redding, CA, USA) in cacodylate buffer for 90 min. The solution was then replaced with 1.5% potassium ferrocyanide (P3289, Sigma-Aldrich) in cacodylate buffer. The samples were then sequentially incubated in filtered 1% thiocarbohydrazide (223220, Sigma-Aldrich) for 45 min at 40°C, 2% aqueous $OsO_4$ for 90 min at room temperature, and 1% uranyl acetate solution overnight at 4°C. The samples were subsequently warmed in a uranyl acetate solution in a 50°C oven for 120 min and then treated with lead aspartate solution for another 120 min at 50°C. After being washed with distilled water, the samples were embedded in a 4% agarose gel. Dehydration was performed using a graded ethanol series followed by two washes with acetone (15 min each). The samples were then incubated in a mixture of acetone and Spurr's resin overnight at room temperature, after which fresh pure resin was applied for an additional 6 h at room temperature. Polymerization was carried out for 48 h at 60°C. For sectioning, ultrathin sections (100 nm thickness) were prepared using an ultramicrotome (Leica) and mounted on silicon wafers (20 mm diameter). For imaging, large-area, high-resolution imaging (35,000×, 1536 × 1024 pixels)

was performed using an Apreo 2S LoVac scanning electron microscope (Thermo Fisher Scientific, Waltham, MA, USA) at the Neuroscience Translational Research Solution Center.

To quantify autophagosome formation and the percentage of abnormal mitochondria in the principal cells of the main segments of the Malpighian tubules, two images per fly were analyzed using ImageJ software. Autophagosome formation was quantified as the number of autophagosomes per $\mu m^2$ of the observed cell body. Eight flies per group were used for quantification. The percentage of abnormal mitochondria (defined as those exhibiting disrupted cristae structures) was calculated based on the total number of mitochondria and those classified as abnormal mitochondria. Five flies per group were used for quantification. The re sults are presented as the mean values with the SDs.

**Western blot analysis**

For immunoblot analysis, the anterior tubules of *Drosophila* males were dissected in PBS. A total of 80-100 pairs of anterior tubules were homogenized and lysed in lysis buffer (20 mM Tris-HCl (pH 7.5), 1 mM EDTA, 5 mM EGTA, 150 mM NaCl, 20 mM NaF, 1% Triton X-100, 1 μg/ml leupeptin, and 1 mM PMSF)[8]. For western blotting of mammalian cells, BEAS-2B cells were lysed in RIPA buffer (50 mM Tris-HCl (pH 7.5), 1 mM EGTA, 50 mM NaCl, 10 mM NaPPi, 50 mM NaF, 5 mM $NaVO_3$, 0.5% Triton X-100, 1 μg/ml Aprotinin, 1 μg/ml leupeptin, 1 μg/ml pepstatinA, 100 μM PMSF, and 1 mM DDT)[9]. All lysates were subjected to western blot analysis as previously described[9]. Anti-SDHB (ab14714) antibodies were purchased from Abcam (Cambridge, UK). Anti-actin (SC-47778) antibodies were purchased from Santa Cruz (Dallas, TX, USA). Anti-ATG5 (#12994) and anti-ATG7 (#8558) antibodies were purchased from Cell Signaling Technology (Danvers, MA, USA). The western blot analyses were repeated at least three times. The band intensities were quantified using ImageJ software (NIH). The results are presented as the mean values with the SDs.

**Analysis of Malpighian tubule width**

To analyze the morphological changes in the Malpighian tubules, we measured the width of the main segments. Seven-day-old naïve male flies were anesthetized on ice and dissected in PBS. The dissected Malpighian tubules were placed in PBS on a glass slide, and images of the main segments were captured with a Leica EZ4E microscope (Leica). To calculate the width of the anterior tubules, measurements of the area and length of the main segment were taken using ImageJ software. The area was divided by the length to compute the width. More than 7 fly samples were used for quantification, and the mean values were calculated. The results are presented as the mean values with the SDs.

**Measurement of longevity**

The longevity of the flies was measured by monitoring survival over time. For longevity analysis, 100 male flies from each group were utilized. The flies were aged and transferred to fresh media every 2-3 days. The number of surviving flies was recorded during each transfer. The results are presented as survival curves or box and whisker plots. In the box and whisker plots, the middle line represents the median, the upper and lower boundaries of the box denote the 25th-75th percentiles, and the whiskers indicate the 5th-95th percentiles, as previously described[10].

**Uric acid analysis**

The uric acid level in the flies was measured as previously described, with slight modifications[10]. To analyze uric acid levels in *Drosophila*, we used the QuantiChrom Uric Acid Assay Kit (DIUA-250, BioAssay Systems, Hayward, CA, USA) according to the manufacturer's instructions. Five flies were homogenized in 100 μl of 0.05% (v/v) Tween-20

via a pestle. Five microlitrers of each sample and uric acid standard were loaded into a 96-well plate, followed by the addition of 200 μl of working reagent. The plate was incubated for 30 minutes at room temperature, and the absorbance was measured at 590 nm via a microplate reader (AMR-100, Allsheng Instruments, Hangzhou, China). The experiments were repeated 5 times for quantification. The results are presented as mean values with the standard errors of the means (S.E.M.).

**Kidney stone analysis**

To analyze kidney stone formation in the Malpighian tubules, we followed a previously described, with slight modifications[10]. HSD was administered to newly eclosed male flies for 3 consecutive weeks, and the flies were dissected in PBS following anesthetization on ice. The dissected Malpighian tubules were placed in PBS on a glass slide, and images were captured via a Leica EZ4E stereomicroscope (Leica). To quantify the tubule stone phenotype, both anterior tubule arms per fly were scored on the scale shown in Supplementary Fig. 4c, and the scores were averaged to obtain a single mean score per fly (ranging from 0 to 4). The results are presented as box and whisker plots. In the plots, the middle line represents the median, the upper and lower boundaries of the box indicate the 25th-75th percentiles, and the whiskers denote the 5th-95th percentiles.

**Survival assay**

The survival rate of the flies was measured as previously described, with slight modifications[9]. For the survival assay, 80 male flies (4 days old) were starved for 6 h, and then transferred to gel media (5% sucrose and 0.5% agar) supplemented with 200 μM PDE701, 5 mM rotenone, or 0.2% DMSO as the vehicle control. Living flies were counted at 24 h intervals and transferred to fresh media every 2 days. The experiment was independently repeated 4

times with 20 flies per group. The results are presented as survival curves, or the survival rates on day 6 are expressed as the mean ± S.E.M.

**Quantitative RT–PCR**

For quantitative real-time PCR analysis, total RNA from 5 flies or 5 x $10^5$ BEAS-2B cells was isolated using an easy-BLUE™ Total RNA Extraction Kit (iNtRON Biotechnology, Seongnam, Korea) according to the manufacturer's instructions, and cDNA was synthesized using TOPscript™ RT DryMIX (Enzynomics, Daejeon, Korea). Quantitative real-time PCR was performed in triplicate using SYBR Green PCR Master Mix (Enzynomics) and an ABI Prism 7500 Real-Time PCR System (Thermo Fisher Scientific, Waltham, MA, USA). rp49 and actin were used as an internal control for all samples, with gene-specific mRNA levels normalized to rp49 in Drosophila and to actin in cells. The mRNA levels were determined using the $2^{-\Delta\Delta CT}$ threshold cycle method. The results are presented as the mean values with the SDs. The primer pairs for qRT-PCR are listed in Supplementary Table 2.

**Statistical analysis**

Differences between two experimental groups were analyzed using Student's *t* test. To compare three or more groups, we used one-way or two-way ANOVA with Šidák correction. The Mann-Whitney U test was used for the statistical analysis of kidney stone scores. The Kruskal-Wallis test followed by Dunn's post hoc correction was used for the statistical analysis of survival rates and the log-rank test was used for the statistical analysis of survival curves. A $p$ value $< 0.05$ was considered statistically significant. All the data were statistically analyzed using Prism 9.0 (GraphPad Software, San Diego, CA, USA).

**References for Methods**


1. Rani, L., Saini, S., Shukla, N., Chowdhuri, D.K. & Gautam, N.K. High sucrose diet induces morphological, structural and functional impairments in the renal tubules of Drosophila melanogaster: A model for studying type-2 diabetes mediated renal tubular dysfunction. *Insect Biochem Mol Biol* **125**, 103441 (2020).

2. Im, S. et al. A novel marine-derived mitophagy inducer ameliorates mitochondrial dysfunction and thermal hypersensitivity in paclitaxel-induced peripheral neuropathy. *Br J Pharmacol* **181**, 4012-4027 (2024).

3. Schellinger, J.N. & Rodan, A.R. Use of the Ramsay Assay to Measure Fluid Secretion and Ion Flux Rates in the Drosophila melanogaster Malpighian Tubule. *J Vis Exp* 53144 (2015).

4. Sun, N. et al. Measuring In Vivo Mitophagy. *Mol Cell* **60**, 685-696 (2015).

5. Kim, Y.Y. et al. Assessment of mitophagy in mt-Keima revealed an essential role of the PINK1-Parkin pathway in mitophagy induction. *Faseb Journal* **33**, 9742-9751 (2019).

6. Albrecht, S.C., Barata, A.G., Grosshans, J., Teleman, A.A. & Dick, T.P. In vivo mapping of hydrogen peroxide and oxidized glutathione reveals chemical and regional specificity of redox homeostasis. *Cell Metab* **14**, 819-829 (2011).

7. Yamaguchi, A. et al. Identifying Therapeutic Agents for Amelioration of Mitochondrial Clearance Disorder in Neurons of Familial Parkinson Disease. *Stem Cell Reports* **14**, 1060-1075 (2020).

8. Choi, S., Lim, D.S. & Chung, J. Feeding and Fasting Signals Converge on the LKB1-SIK3 Pathway to Regulate Lipid Metabolism in Drosophila. *PLoS Genet* **11**, e1005263 (2015).

9. Jeong, D.J. et al. The Mst1/2-BNIP3 axis is required for mitophagy induction and neuronal viability under mitochondrial stress. *Exp Mol Med* **56**, 674-685 (2024).

10. van Dam, E. et al. Sugar-Induced Obesity and Insulin Resistance Are Uncoupled from Shortened Survival in Drosophila. *Cell Metab* **31**, 710-725 e717 (2020).

**Supplementary Table 1. Genotypes of *Drosophila***

| Figure | Genotype |
|---|---|
| 1b | *w<sup>1118</sup>* |
| 1b,d,e,g | *UAS-dmt-Keima/+; da-Gal4/+* |
| 1f | *w<sup>1118</sup>/Y; UAS-dmt-Keima/+; da-Gal4/+*<br>*UAS-ATG5 RNAi/Y; UAS-dmt-Keima/+; da-Gal4/+* |
| 2b,c | *esg-Gal4/UAS-dmt-Keima* |
| 2d,e | *UAS-dmt-Keima/+; c42-Gal4/+* |
| 2f,g | *tsh-Gal4/UAS-dmt-Keima* |
| 3a | *w<sup>1118</sup>/Y; tubP-Gal80<sup>ts</sup>/+; c42-Gal4, UAS-dmt-Keima/+*<br>*UAS-ATG5 RNAi/Y; tubP-Gal80<sup>ts</sup>/+; c42-Gal4, UAS-dmt-Keima/+*<br>*tubP-Gal80<sup>ts</sup>/UAS-ULK1 RNAi; c42-Gal4, UAS-dmt-Keima/+*<br>*tubP-Gal80<sup>ts</sup>/UAS-Parkin RNAi; c42-Gal4, UAS-dmt-Keima/+* |
| 3b-d | *w<sup>1118</sup>/Y; tubP-Gal80<sup>ts</sup>/+; c42-Gal4/+* |
| 3b | *UAS-ATG5 RNAi/Y; tubP-Gal80<sup>ts</sup>/+; c42-Gal4/+* |
| 3c | *tubP-Gal80<sup>ts</sup>/UAS-ULK1 RNAi; c42-Gal4/+* |
| 3d | *tubP-Gal80<sup>ts</sup>/UAS-Parkin RNAi; c42-Gal4/+* |
| 4b | *UAS-dmt-Keima/+; c42-Gal4/+* |
| 4c,f,g | *w<sup>1118</sup>* |
| 4d | *UAS-mito-roGFP2-Orp1/+; c42-Gal4/+* |
| 4e | *UAS-mitoHAGFP/+; c42-Gal4/+* |
| 5b | *UAS-dmt-Keima/+; c42-Gal4/+* |
| 5c | *UAS-mito-roGFP2-Orp1/+; c42-Gal4/+* |
| 5d,e | *w<sup>1118</sup>* |
| 5f | *w<sup>1118</sup>/Y; tubP-Gal80<sup>ts</sup>, UAS-mito-roGFP2-Orp1/+; c42-Gal4/+*<br>*tubP-Gal80<sup>ts</sup>, UAS-mito-roGFP2-Orp1/+; c42-Gal4/UAS-Sod1* |
| 5g | *w<sup>1118</sup>/Y; tubP-Gal80<sup>ts</sup>/+; c42-Gal4, UAS-dmt-Keima/+*<br>*tubP-Gal80<sup>ts</sup>/+; c42-Gal4, UAS-dmt-Keima/UAS-Sod1* |
| 5h | *w<sup>1118</sup>/Y; tubP-Gal80<sup>ts</sup>/+; c42-Gal4/+*<br>*tubP-Gal80<sup>ts</sup>/+; c42-Gal4/UAS-Sod1* |
| 6b | *UAS-dmt-Keima/+; c42-Gal4/+* |
| 6c | *UAS-mito-roGFP2-Orp1/+; c42-Gal4/+* |
| 6d | *UAS-mitoHAGFP/+; c42-Gal4/+* |
| 6e-h | *w<sup>1118</sup>* |
| 7a | *UAS-dmt-Keima/+; c42-Gal4/+* |
| 7b | *w<sup>1118</sup>* |
| 7c,e | *w<sup>1118</sup>/Y; tubP-Gal80<sup>ts</sup>/+; c42-Gal4, UAS-dmt-Keima/+* |
| 7c | *UAS-ATG5 RNAi/Y; tubP-Gal80<sup>ts</sup>/+; c42-Gal4, UAS-dmt-Keima/+* |
| 7d,f | *w<sup>1118</sup>/Y; tubP-Gal80<sup>ts</sup>/+; c42-Gal4/+* |
| 7d | *UAS-ATG5 RNAi/Y; tubP-Gal80<sup>ts</sup>/+; c42-Gal4/+* |
| 7e | *tubP-Gal80<sup>ts</sup>/+; c42-Gal4, UAS-dmt-Keima/UAS-ATG7 RNAi* |

| | |
|---|---|
| 7f | *tubP-Gal80$^{ts}$/+; c42-Gal4/UAS-ATG7 RNAi* |
| Supplementary Figuers | |
| s1a-c | *UAS-mt-Keima/+; da-Gal4/+*<br>*UAS-dmt-Keima/+; da-Gal4/+* |
| s1d | *UAS-mitoQC/+; da-Gal4/+* |
| s2a | *w$^{1118}$*<br>*UAS-dmt-Keima/+; c42-Gal4/+*<br>*esg-Gal4/UAS-dmt-Keima*<br>*tsh-Gal4/UAS-dmt-Keima* |
| s2b | *esg-Gal4/UAS-dmt-Keima*<br>*UAS-dmt-Keima/+; c42-Gal4/+* |
| s2c | *w$^{1118}$/Y; esg-Gal4/UAS-dmt-Keima*<br>*UAS-ATG5 RNAi/Y; esg-Gal4/UAS-dmt-Keima* |
| s2d | *w$^{1118}$/Y; UAS-dmt-Keima/+; c42-Gal4/+*<br>*UAS-ATG5 RNAi/Y; UAS-dmt-Keima/+; c42-Gal4/+* |
| s2e | *w$^{1118}$/Y; UAS-mitoQC/+; c42-Gal4/+*<br>*UAS-ATG5 RNAi/Y; UAS-mitoQC/+; c42-Gal4/+* |
| s3a-c | *w$^{1118}$/Y; ; da-Gal4/+* |
| s3a | *UAS-ATG5 RNAi/Y; ; da-Gal4/+* |
| s3b | *UAS-ULK1 RNAi/+; da-Gal4/+* |
| s3c | *UAS-Parkin RNAi/+; da-Gal4/+* |
| s3d | *UAS-dmt-Keima/+; da-Gal4/+*<br>*da-Gal4/UAS-dmt-Keima* |
| s4a | *UAS-mitoQC/+; da-Gal4/+* |
| s4b | *UAS-mitoHAGFP/+; c42-Gal4/+* |
| s4c-e | *w$^{1118}$* |
| s5a | *w$^{1118}$/Y; tubP-Gal80$^{ts}$/+; c42-Gal4, UAS-dmt-Keima/+*<br>*tubP-Gal80$^{ts}$/UAS-AMPKα.WT; c42-Gal4, UAS-dmt-Keima/+* |
| s5b | *w$^{1118}$/Y; tubP-Gal80$^{ts}$/+; c42-Gal4, UAS-dmt-Keima/+*<br>*tubP-Gal80$^{ts}$/+; c42-Gal4, UAS-dmt-Keima/UAS-Tor RNAi* |
| s5c | *w$^{1118}$/Y; ; da-Gal4/+*<br>*da-Gal4/UAS-SOD1* |
| s5d | *w$^{1118}$/Y; ; da-Gal4/+*<br>*UAS-AMPKα.WT /+; da-Gal4/+* |
| s5e | *w$^{1118}$/Y; ; da-Gal4/+*<br>*da-Gal4/UAS-Tor RNAi* |
| s6a,e | *UAS-dmt-Keima/+; c42-Gal4/+* |
| s6b | *UAS-mitoQC/+; c42-Gal4/+* |
| s6c | *UAS-mitoHAGFP/+; c42-Gal4/+* |
| s6d,f,g | *w$^{1118}$* |
| s7a | *w$^{1118}$/Y; tubP-Gal80$^{ts}$/+; c42-Gal4, UAS-dmt-Keima/+*<br>*tubP-Gal80$^{ts}$/UAS-Parkin RNAi; c42-Gal4, UAS-dmt-Keima/+* |
| s7b | *w$^{1118}$/Y; tubP-Gal80$^{ts}$/+; c42-Gal4, UAS-dmt-Keima/+*<br>*tubP-Gal80$^{ts}$/UAS-AMPKα RNAi; c42-Gal4, UAS-dmt-Keima/+* |
| s7c | *w$^{1118}$/Y; ; da-Gal4/+*<br>*da-Gal4/UAS-ATG7 RNAi* |

| | |
|---|---|
| s7d | *$w^{1118}$/Y; ; da-Gal4/+*<br>*UAS-AMPKα RNAi/+; da-Gal4/+* |
| s8a,b | *$w^{1118}$* |

**Supplementary Table 2. List of primers for qRT-PCR**

| primer | Sequence (5’ to 3’) |
|---|---|
| For *Drosophila* | |
| rp49-F | GCTTCAAGATGACCATCCGCCC |
| rp49-R | GGTGCGCTTGTTCGATCCGTAAC |
| ATG5-F | GCACGCACGGCATTGATCTACA |
| ATG5-R | GCCCTGGGATTTGCTGGAAT |
| ULK1-F | ACCATGAACGCCAAATACCG |
| ULK1-R | ATGCATTCGCCTTCTGCAAT |
| parkin-F | GGGTCCACGGTGTACTCGC |
| parkin-R | TACCGTGTTGGTTTTCCCCTGC |
| SOD1-F | CACCGACTCCAAGATTACGCTC |
| SOD1-R | AATGCCAATAACGCCGCACC |
| AMPK-F | CCGGCAAGTTCTCGAAGAT |
| AMPK-R | GAGTAAGGCTCTCCATGATGAC |
| Tor-F | CTAGCCAATCGGAGGTGTCC |
| Tor-R | CGACCCTCCTCCTCAACAC |
| ATG7-F | TTTTGCCTCACTCCATCCGTGG |
| ATG7-R | ATCCTCGTCGCTATCGGACAT |
| For cells | |
| Actin-F | CATGTACGTTGCTATCCAGGC |
| Actin-R | CTCCTTAATGTCACGCACGAT |
| ATG5-F | AAAGATGTGCTTCGAGATGTGT |
| ATG5-R | CACTTTGTCAGTTACCAACGTCA |
| ATG7-F | CAGTTTGCCCCTTTTAGTAGTGC |
| ATG7-R | CCAGCCGATACTCGTTCAGC |

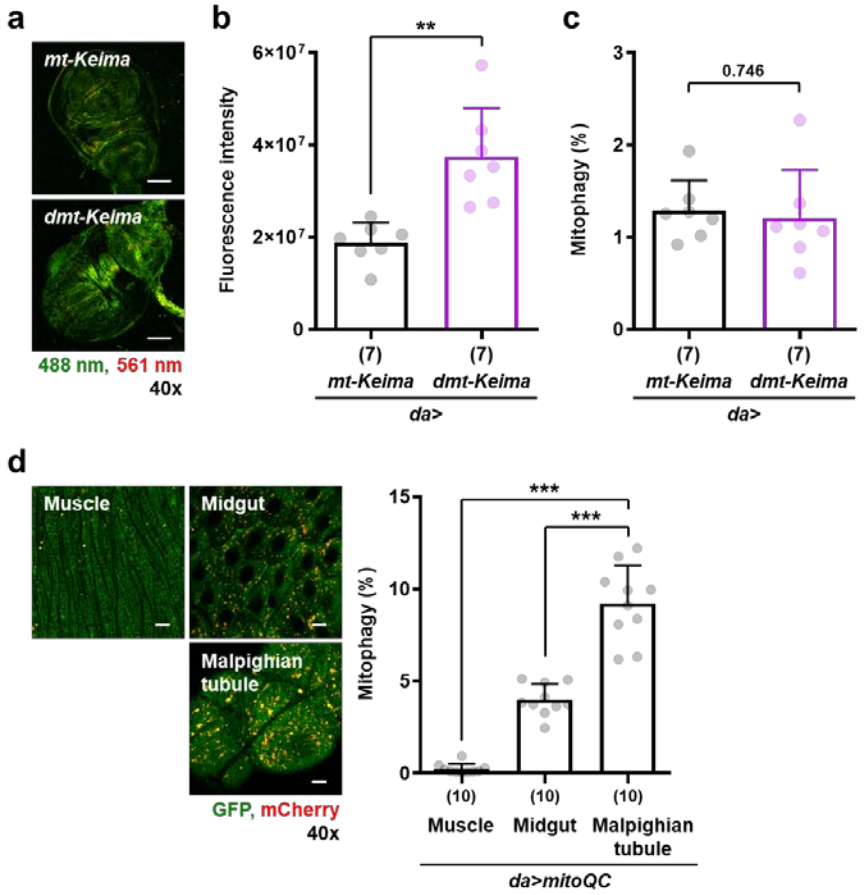


**Supplementary Fig. 1. Assessment of mitophagy in dmt-Keima *Drosophila*.**

**a-c.** Representative mt-Keima or dmt-Keima fluorescence images **(a),** quantitative fluorescence intensity (**b**) and quantitative analysis of mitophagy (**c**) in wing discs from 3rd instar larvae carrying *da>mt-Keima* or *da>dmt-Keima*. The data are shown as means ± SDs and quantitative fluorescence intensities (**b**) or quantified mitophagy levels (**c**) of $n$ = 7 individual larvae. **d.** Assessment of the mitophagy level in various fly tissues from adult male flies harboring *da>mitoQC*. Representative mitoQC fluorescence images (left) and quantitative analysis of mitophagy (right). Scale bars, 10 µm. dmt-Keima fluorescence was imaged at 488 nm (green) and 561 nm (red); mitophagy levels were quantified based on the red/green fluorescence ratio, which reflects mitochondrial acidification. 40x indicates the magnification of the objective lense used for confocal imaging. The numbers in parentheses indicate $n$. The data are shown as means ± SDs ($n$ = 10). Significance was determined by Student's $t$ test (**b, c**) or one-way ANOVA with Šidák correction (**d**). The number above the bars is the $p$ value. ** $p$ < 0.01; *** $p$ < 0.001.

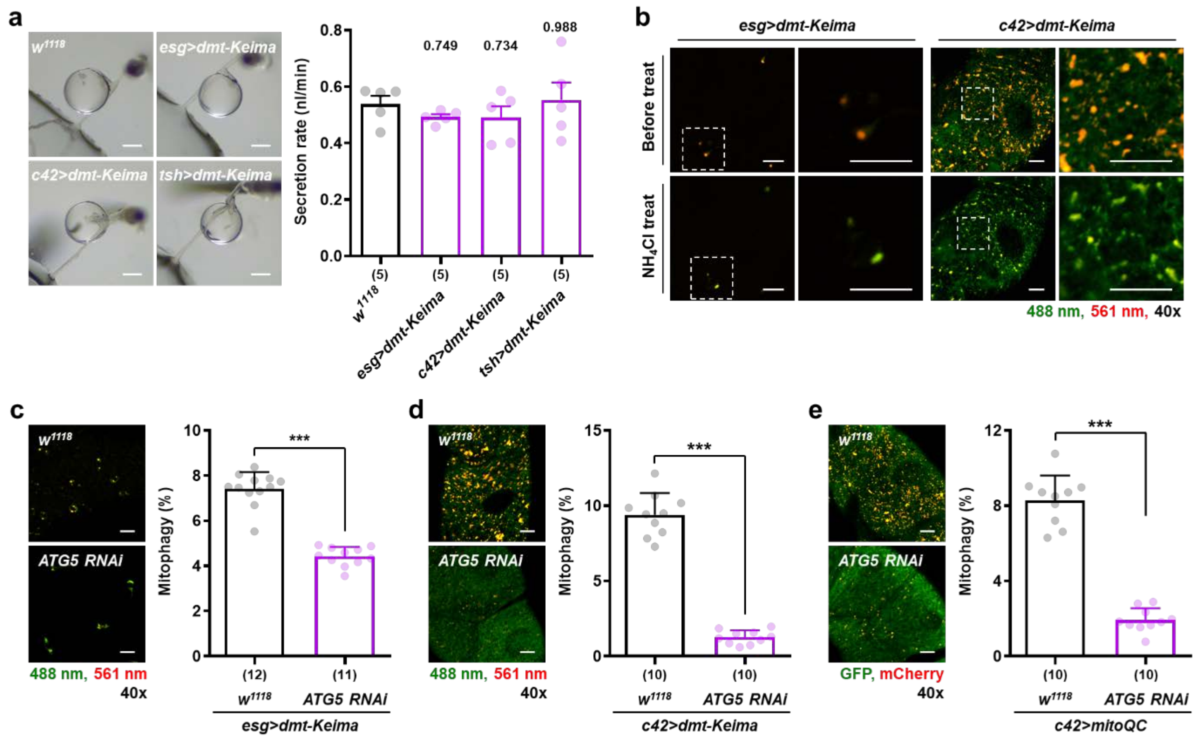


**Supplementary Fig. 2. The mitophagy level depends on the cell type and structural location within the Malpighian tubules of *Drosophila.***

**a.** Representative images of fluid droplets from the Malpighian tubules of male flies of the indicated genotypes (left). Fluid secretion rates for Malpighian tubules from each fly strain (right). The data are shown as means ± S.E.M. (5 biological replicates per group). Scale bars, 200 μm. **b.** Effects of $NH_4Cl$ treatment on mt-Keima fluorescence. Renal stem cells in the ureter (left) or principal cells in the main segment (right) of the Malpighian tubules of male flies of the indicated genotypes were treated with $NH_4Cl$ (50 mM). The boxed regions are presented in an enlarged format in the lower panel for a more detailed view. Scale bars, 10 μm. **c.** Representative dmt-Keima fluorescence images (left) and quantitative mitophagy levels (right) of renal stem cells in the ureter of Malpighian tubules from control flies (*esg>dmt-Keima*, $w^{1118}$) or ATG5 RNAi (*esg>dmt-Keima*, *ATG5 RNAi*). The data are shown as means ± SDs ($n$ = 11-12). Scale bars, 10 μm. **d, e.** Representative dmt-Keima or mitoQC fluorescence images (left) and quantitative mitophagy levels (right) of principal cells in the main segment of Malpighian tubules from control flies or ATG5 RNAi. dmt-Keima fluorescence was imaged at 488 nm (green) and 561 nm (red); mitophagy levels were quantified based on the red/green fluorescence ratio, which reflects mitochondrial acidification. 40x indicates the magnification of the objective lense used for confocal imaging. The numbers in parentheses indicate $n$. The

data are shown as means ± SDs ($n = 10$). Scale bars, 10 μm. Significance was determined by one-way ANOVA with Šidák correction (**a**) or Student's *t* test (**c-e**). The numbers above the bars are the *p* values. *** $p < 0.001$.

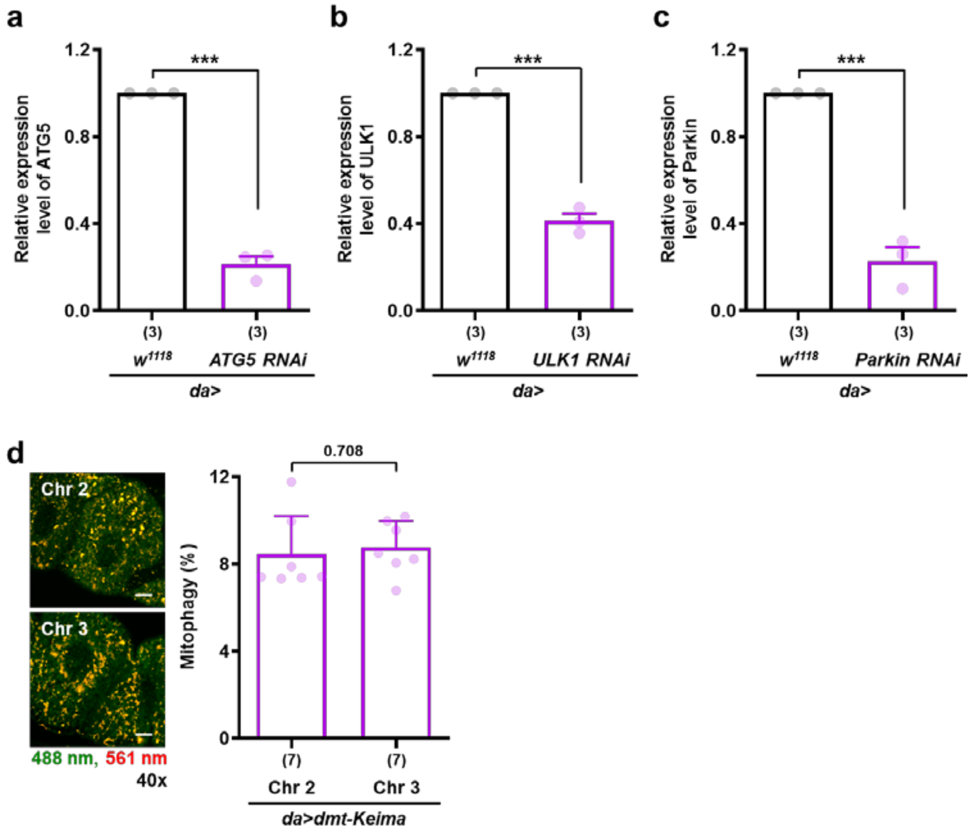


**Supplementary Fig. 3. Verification of gene knockdown and mitophagy levels in dmt-Keima flies.**

**a-c.** mRNA transcript levels in the whole bodies of 5 male flies subjected to each RNAi. To efficiently validate the knockdown efficiency, RNAi constructs were expressed under the control of *da-GAL4*, allowing robust detection of transcript suppression from whole-body RNA. The data are shown as means ± SDs (3 biological replicates per group). **d.** Representative fluorescence images of dmt-Keima on chromosome 2 (UAS-dmt-Keima/+; da-Gal4/+) (Chr2) and chromosome 3 (da-Gal4/UAS-dmt-Keima) (Chr3) are shown on the left. Mitophagy was quantified by the red-to-green fluorescence ratio of dmt-Keima (561/488 nm), which indicates mitochondrial acidification. The results of the quantitative analysis are shown on the right as means ± SDs ($n$ = 7). Scale bars, 10 μm. 40x indicates the magnification of the objective lense used for confocal imaging. The numbers in parentheses indicate $n$. Significance was determined by Student's $t$ test. The number above the bars is the $p$ value. *** $p < 0.001$.

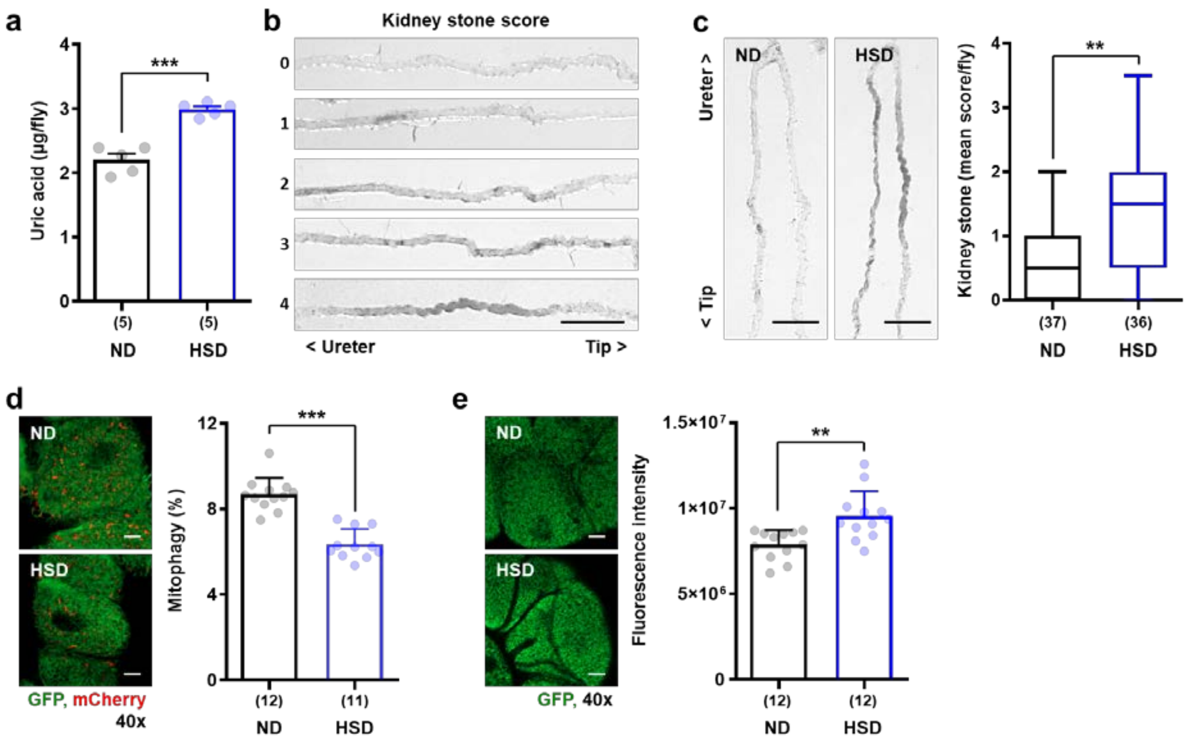


**Supplementary Fig. 4. A high-sugar diet leads to mitochondrial impairment and functional anomalies in Malpighian tubules.**

**a**. Uric acid levels were analyzed in the whole body after the administration of ND or HSD for 7 days to newly eclosed male files (*w*$^{1118}$). The data are shown as means ± SEMs (5 biological replicates per group). **b.** Standard figures for scoring kidney stones in Malpighian tubules. **c**. Box and whisker plot for the kidney stone scores of *n* = 36-37 *w*$^{1118}$ male flies. In the box and whisker plot, the middle line represents the median, the upper and lower boundaries of the box denote the 25th-75th percentiles, and the whiskers indicate the 5th-95th percentiles. Scale bars, 200 µm. **d.** Representative mitoQC fluorescence images (left) and quantitative mitophagy levels (right) of principal cells in the main segment from 7-day-old adult male flies carrying *c42>mitoQC* under ND or HSD conditions. The data are shown as the means ± SDs (*n* = 11-12). Scale bars, 10 µm. **e**. Representative mitoHAGFP fluorescence images (left) and quantified fluorescence intensity (right) of principal cells in the main segment from 7-day-old adult male flies carrying *c42>mitoHAGFP* under ND or HSD conditions. The data are shown as means ± SDs (*n* = 12). Scale bars, 10 µm. 40x indicates the magnification of the objective lense used for confocal imaging. The numbers in parentheses indicate *n*. The numbers in parentheses indicate *n*. Significance was determined by Student's *t* test (**a, d, e**) or Mann-Whitney test (**d**). ** $p < 0.01$; *** $p < 0.001$.

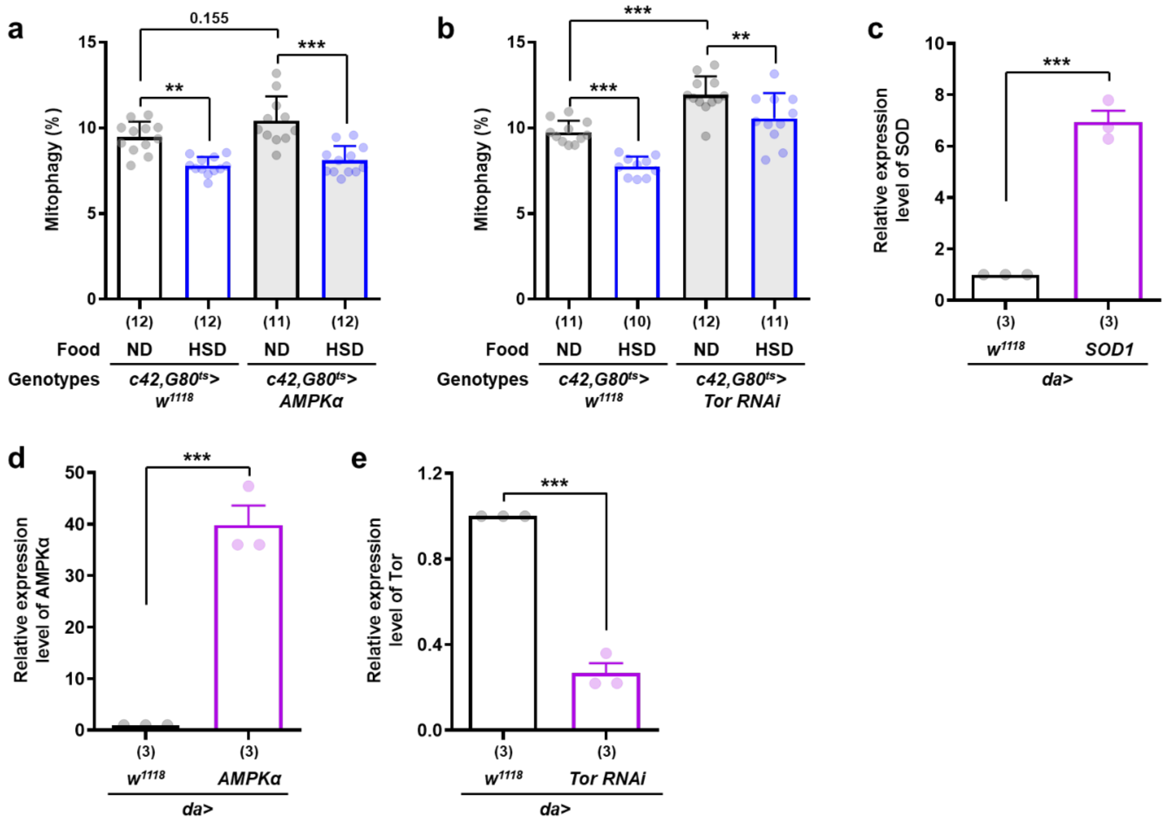


**Supplementary Fig. 5. HSD induces a decrease in mitophagy in Malpighian tubules, followed by subsequent functional abnormalities.**

**a, b.** Quantification of mitophagy in principal cells in the main segment from control, AMPK*α* overexpressing (**a**), or Tor RNAi (**b**) male flies fed an ND or HSD for 7 days. The data are shown as means ± SDs ($n$ = 10-12). **c-e.** mRNA transcript levels from the whole body of 5 males under each overexpression (**c, d**) or RNAi (**e**) condition. The data are shown as means ± SDs (3 biological replicates per group). The data are shown as means ± SDs ($n$ = 10-12). The numbers in parentheses indicate $n$. Significance was determined by two-way ANOVA with Šidák correction (**a, b**) or Student's $t$ test (**c-e**). The number above the bars is the $p$ value. ** $p < 0.01$; *** $p < 0.001$.

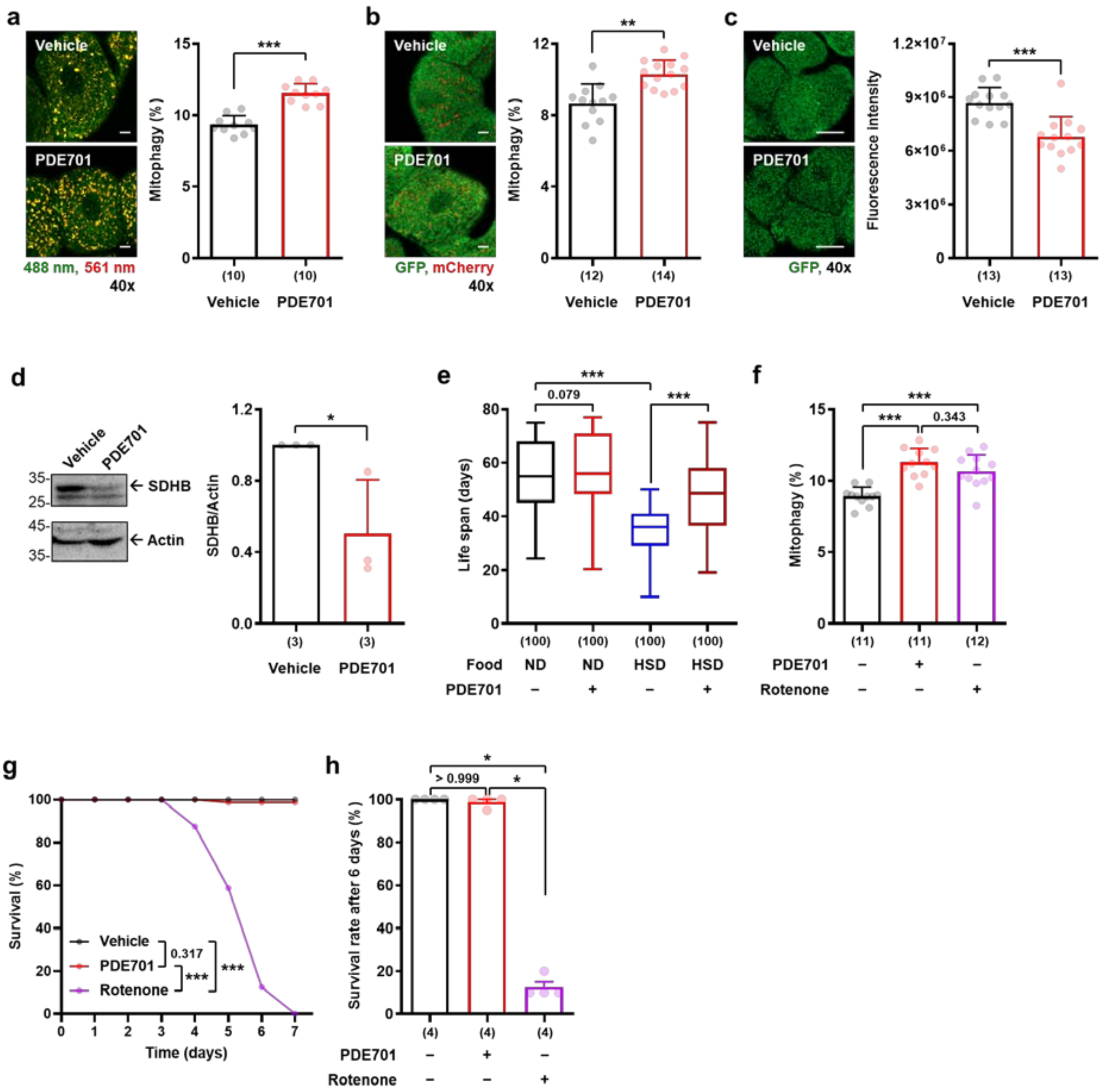

**Supplementary Fig. 6. Verification of mitophagy induction in Malpighian tubules upon PDE701 administration.**

**a, b.** Representative fluorescence images (left) and quantitative mitophagy levels (right) of principal cells in the main segment from 7-day-old adult male flies carrying *c42>dmt-Keima* (**a**) or *c42>mitoQC* (**b**) with or without PDE701 treatment for 2 days. Mitophagy was assessed by the red-to-green fluorescence ratio of dmt-Keima (561/488 nm), which indicates mitochondrial acidification. The data are shown as means ± SDs ($n$ = 10-14). Scale bars, 10 μm. **c.** Representative mitoHAGFP fluorescence images (left) and quantified fluorescence intensity (right) of principal cells in the main segment from 7-day-old adult male flies carrying

*c42>mitoHAGFP* under each condition. The data are shown as means ± SDs ($n$ = 13). Scale bars, 10 μm. 40x indicates the magnification of the objective lense used for confocal imaging. **d**. Representative immunoblot images (left) and quantified protein levels of SDHB (right) of the Malpighian tubules from 7-day-old $w^{1118}$ male flies under each condition. The results from three biological replicates are shown as the mean ± SD. **e.** Box and whisker plot for the lifespan of $n$ = 100 $w^{1118}$ male flies under the indicated conditions. In the box and whisker plot, the middle line represents the median, the upper and lower boundaries of the box denote the 25th-75th percentiles, and the whiskers indicate the 5th-95th percentiles. **f.** Quantification of mitophagy in principal cells of adult male flies carrying *c42>dmt-Keima* with 200 μM PDE701 or 5 mM rotenone for 2 days prior to subsequent assays. The data are shown as means ± SDs ($n$ = 11-12). **g.** Survival curve of $n$ = 80 $w^{1118}$ male flies treated with PDE701 or rotenone. **h.** Survival rate on day 6 following PDE701 or rotenone treatment. The data are shown as means ± SEMs (4 biological replicates per group). The numbers in parentheses indicate *n*. Significance was determined by Student's *t* test (**a-d**), the log-rank test (**e, g**), one-way ANOVA with Šidák correction (**f**), or the Kruskal-Wallis test with Dunn's post hoc correction (**h**). The numbers above the bars are the *p* values. * $p < 0.05$; ** $p < 0.01$; *** $p < 0.001$.

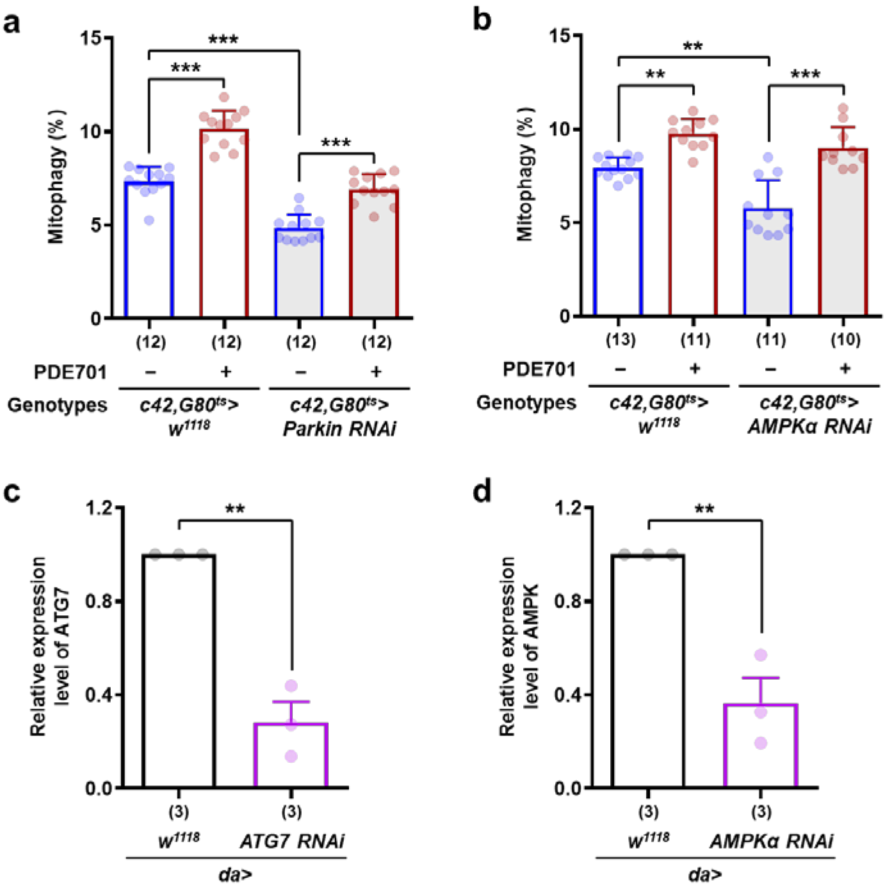


**Supplementary Fig. 7. PDE701 stimulates mitophagy independently of AMPKα and Parkin in principal cells.**

**a, b**. Quantification of mitophagy in principal cells in the main segment from control, Parkin RNAi (a) or AMPKα RNAi (b) male flies reared on HSD and treated with PDE701 following the experimental design shown in Fig. 6a. The data are shown as means ± SDs (n = 10-13). **c, d.** mRNA transcript levels from the whole body of 5 males under each RNAi. The data are shown as means ± SDs (3 biological replicates per group). The numbers in parentheses indicate *n*. Significance was determined by two-way ANOVA with Šidák correction (**a, b**) or Student's t test (**c, d**). ** $p < 0.01$; *** $p < 0.001$.

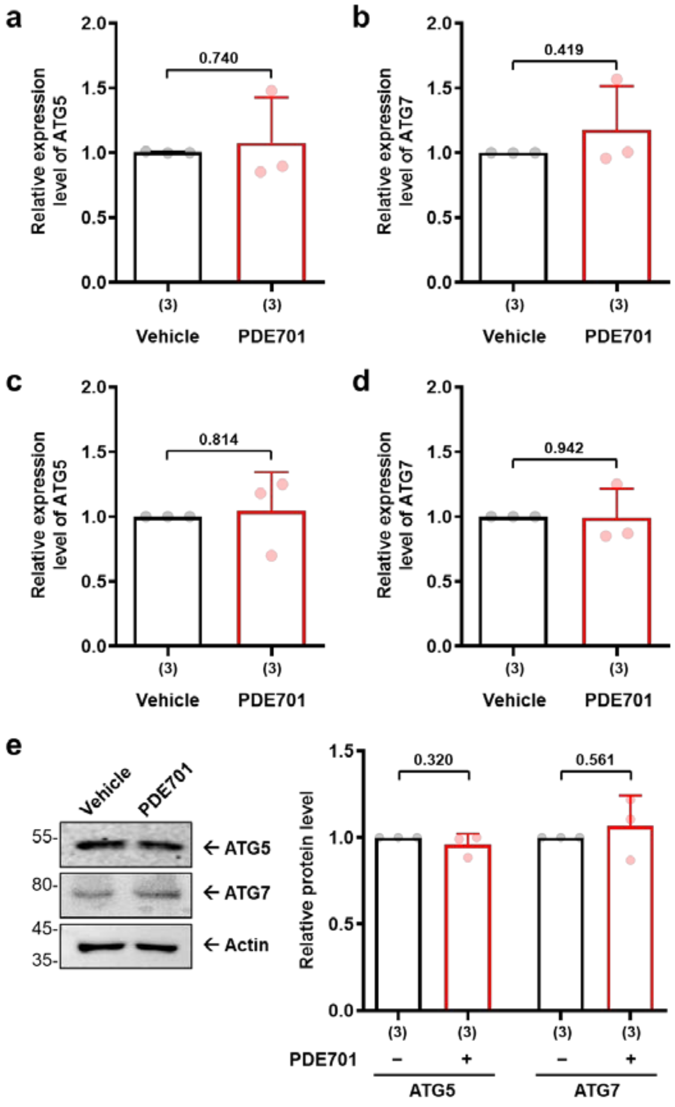


**Supplementary Fig. 8. ATG5 and ATG7 expression levels remain unchanged following PDE701 treatment in *Drosophila* and BEAS-2B cells.**

**a, b**. mRNA levels of ATG5 and ATG7 in whole body samples from five $w^{1118}$ males with or without PDE701 treatment for 2 days. **c, d**. mRNA levels of ATG5 and ATG7 in BEAS-2B cells treated with or without 6 μM PDE701 for 24 h. **e**. Representative immunoblot images (left) and quantification of ATG5 and ATG7 protein levels (right) in BEAS-2B cells under the same condition. The results from three biological replicates are shown as the mean ± SD. The numbers in parentheses indicate *n*. Significance was determined by Student's *t* test. The numbers above the bars are the *p* values.
